# Beyond Technologies of Electroencephalography-Based Brain-Computer Interfaces: A Systematic Review From Commercial and Ethical Aspects

**DOI:** 10.3389/fnins.2020.611130

**Published:** 2020-12-17

**Authors:** Cesar Augusto Fontanillo Lopez, Guangye Li, Dingguo Zhang

**Affiliations:** ^1^KU-Leuven Center for IT & IP Law, KU-Leuven, Leuven, Belgium; ^2^The Robotics Institute, School of Mechanical Engineering, Shanghai Jiao Tong University, Shanghai, China; ^3^The Department of Electronic and Electrical Engineering, University of Bath, Bath, United Kingdom

**Keywords:** electroencephalography, brain-computer interface, commercial aspects, ethical aspects, EEG

## Abstract

The deployment of electroencephalographic techniques for commercial applications has undergone a rapid growth in recent decades. As they continue to expand in the consumer markets as suitable techniques for monitoring the brain activity, their transformative potential necessitates equally significant ethical inquiries. One of the main questions, which arises then when evaluating these kinds of applications, is whether they should be aligned or not with the main ethical concerns reported by scholars and experts. Thus, the present work attempts to unify these disciplines of knowledge by performing a comprehensive scan of the major electroencephalographic market applications as well as their most relevant ethical concerns arising from the existing literature. In this literature review, different databases were consulted, which presented conceptual and empirical discussions and findings about commercial and ethical aspects of electroencephalography. Subsequently, the content was extracted from the articles and the main conclusions were presented. Finally, an external assessment of the outcomes was conducted in consultation with an expert panel in some of the topic areas such as biomedical engineering, biomechatronics, and neuroscience. The ultimate purpose of this review is to provide a genuine insight into the cutting-edge practical attempts at electroencephalography. By the same token, it seeks to highlight the overlap between the market needs and the ethical standards that should govern the deployment of electroencephalographic consumer-grade solutions, providing a practical approach that overcomes the engineering myopia of certain ethical discussions.

## Introduction

Electroencephalography (EEG) is one of the most widespread neuroimaging techniques. It is not only a rapidly developing area of neuroscience research but also a technology which attracts a considerable amount of attention and investment.

Some of the keys to the success of EEG are their towering advantages among other brain-imaging techniques. Thus, EEG offers superior safety, portability, temporal resolution and cost-effectiveness than other non-invasive methods, such as functional magnetic resonance imaging (fMRI), magnetoencephalography (MEG), or positron emission tomography (PET) (Akcakaya et al., 2014). These advantages have made EEG a widely accepted tool by the scientific community and the private sector for neuroscience research and applications.

EEG uses are extremely wide-ranging and have undergone profound changes in recent years. Initially, EEG was adopted to translate users' intentions by classifying their voluntary brain activity to actively monitor or control external devices. These applications have been called active Brain-Computer Interfaces (aBCI) and are ordinarily confined in the biomedical field for replacing, restoring, enhancing, supplementing, or improving natural central nervous system (CNS) output (Zander et al., 2010; Burwell et al., 2017; Wolpaw et al., 2020). However, at a later stage, EEG applications have evolved from their original scientific purpose to passively decode cognitive and emotional states of users' spontaneous brain activity. These new systems have extended the traditional notions of aBCI applications to passive Brain-Computer Interfaces (pBCI) (Zander and Kothe, 2011; Blankertz et al., 2016; Arico et al., 2017; Aricò et al., 2018). In turn, pBCI applications have enhanced the business prospects of EEG because of their commercial value as tracking tool solutions that can be exploited in consumer markets.

During this transition, the technological improvements and implications of EEG have been profoundly considered by a wide range of literature reviews (Jackson and Bolger, 2014; Marzbani et al., 2016; Enriquez-Geppert et al., 2017). Yet the vast majority of these studies have been conducted from an engineering standpoint. Since the main proposed EEG use is as an assistive technology, most of the studies have mainly concentrated on neurofeedback improvements as it primarily relates to biomedical applications of aBCIs. In contrast, little research has been done into the intersection of the current state-of-the-art in commercial applications of EEG and their ethical concerns.

By taking into account the major market applications of EEG, the current scoping review outlines the efforts which have been made in heterogeneous business sectors, and provides illustrative examples of existing EEG projects and business initiatives. On this basis, the review also identifies the most commonly cited ethical issues that have been acknowledged in the existing literature, and elaborates upon the various postures which could be adopted with regards to the present development of EEG. Most importantly, the final goal of this review is to provide better insights about the existing opportunities and challenges for the transition into a BCI society, where the deployment of EEG technologies is carried out with respect for social ethical frameworks.

## Methods

A comprehensive literature review was performed by applying the methodology proposed by Levac et al. (2010), Burwell et al. (2017), as an update of Arksey and O'Malley's original method of literature review (Arksey and O'Malley, 2005). The review framework includes the original stages enumerated by the authors: (a) identifying the research question, (b) identifying relevant studies, (c) study selection, (d) charting the data and collating, summarizing, and reporting the results, and (e) consultation.

### Identifying the Research Question

The research goal is to analyze the current market applications of EEG in order to confront them with the dominant literature on ethics. The clarification of this gap could provide a pragmatic approach of the present ethical debate and inform recommendations for future research.

### Identifying Relevant Studies

**Figure 1:**
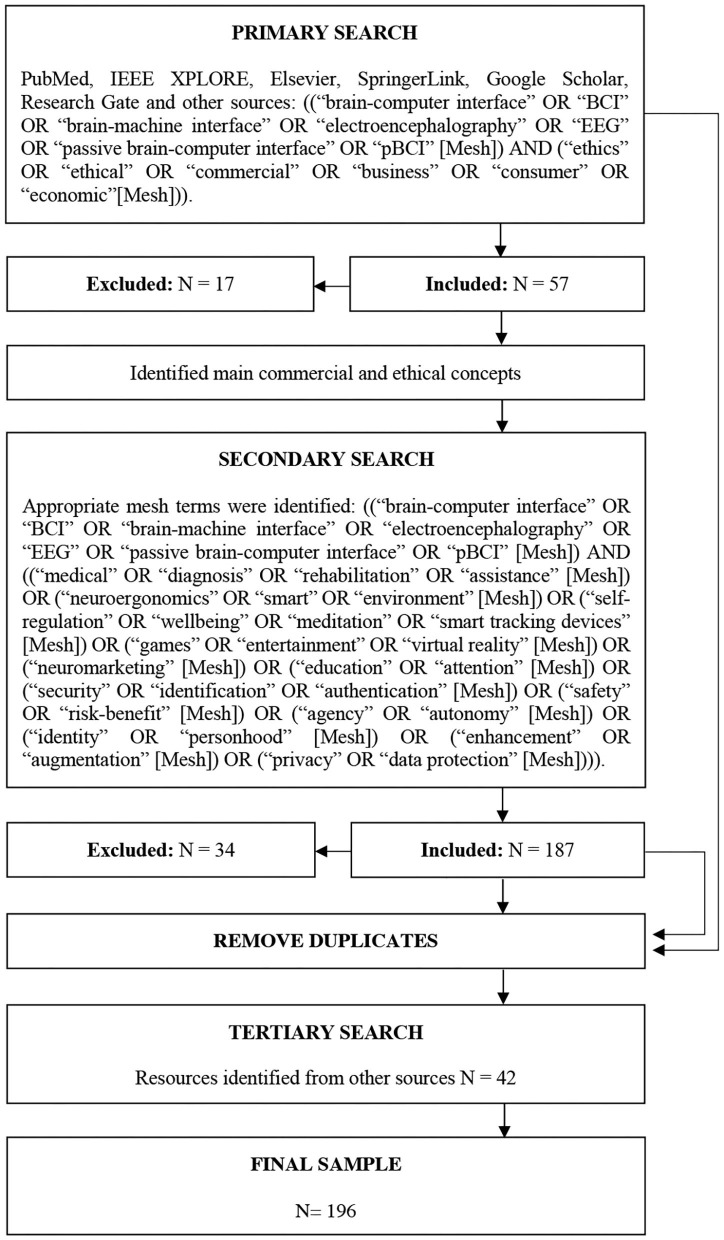
Search strategy.

The primary searches focused on different bibliographic databases such as (a) PubMed (b) IEEE XPLORE, (c) Elsevier, (d) SpringerLink, (e) Google Scholar, (f) ResearchGate, and (g) other sources (Figure 1). These databases were chosen due to their range spectrum, specifically regarding the commercial and ethical considerations of EEG. Several searches were conducted by using the keywords related to the domains of commercial applications and ethical issues of EEG in general.

The primary searches occurred during August 2018. The keywords used for the primary searches were ((“brain-computer interface” OR “BCI” OR “brain-machine interface” OR “electroencephalography” OR “EEG” OR “passive brain-computer interface” OR “pBCI” [Mesh]) AND (“ethics” OR “ethical” OR “commercial” OR “business” OR “consumer” OR “economic” [Mesh])).

Articles were included if they (1) were written in English, German, Spanish or French (2) presented conceptual discussions or empirical findings on ethics or commercial aspects of BCI, and (3) were especially related to EEG technologies. After applying these criteria, 57 articles remained from the primary search (*N* = 57).

### Selecting Studies for Inclusion

A list of different EEG applications as well as a list of topics frequently discussed in the ethics literature were identified from the primary search. Following the primary search, varying keywords were generated. Then, secondary targeted searches were performed to include articles that were framed in terms of a specific topic within the domains of consumer-grade devices and ethics. The secondary searches occurred between September and December 2018. The keywords used for the secondary targeted searches were ((“brain-computer interface” OR “BCI” OR “brain-machine interface” OR “electroencephalography” OR “EEG” OR “passive brain-computer interface” OR “pBCI” [Mesh]) AND ((“medical” OR “diagnosis” OR “rehabilitation” OR “assistance” [Mesh]) OR (“neuroergonomics” OR “smart” OR “environment” [Mesh]) OR (“self-regulation” OR “wel-lbeing” OR “meditation” OR “smart tracking devices” [Mesh]) OR (“games” OR “entertainment” OR “virtual reality” [Mesh]) OR (“neuromarketing” [Mesh]) OR (“education” OR “attention” [Mesh]) OR (“security” OR “identification” OR “authentication” [Mesh]) OR (“safety” OR “risk-benefit” [Mesh]) OR (“agency” OR “autonomy” [Mesh]) OR (“identity” OR “personhood” [Mesh]) OR (“enhancement” OR “augmentation” [Mesh]) OR (“privacy” OR “data protection” [Mesh]))). After applying the same inclusion and exclusion criteria as the primary searches, the secondary searches yielded 187 articles. Once duplicate articles from the primary and secondary searches were excluded, there remained a total of 154 articles.

Following the primary and secondary searches, further relevant sources (*N* = 42) were consulted in tertiary searches, which included, among others, several articles highlighted in the consultation phase. No duplicates were found with the previous searches. The final sample remained *N* = 196 articles.

### Charting the Data and Collating, Summarizing, and Reporting the Results

From the primary and secondary searches (*N* = 154), the specific commercial and ethical issues were identified and the content was extracted. DZ reviewed the extracted content and provided feedback on its organization. Thus, the main conclusions within the commercial and ethical realms were presented in a narrative fashion.

### Consultation

An external assessment of the outcomes of this review was conducted in consultation with three experts in some of the topic areas such as biomedical engineering, biorobotics and biomechatronics, and neuroscience. Feedback was considered to revise the manuscript as well as to ponder valuable insights that the scoping review alone would not have identified (Daudt et al., 2013).

Finally, the organization of the paper remains as follows: in the first section, the results of the review will be presented and discussed from a commercial and ethical standpoint. In the second section, the conclusions of the review will be elaborated upon.

## Commercial Aspects

**Figure 2:**
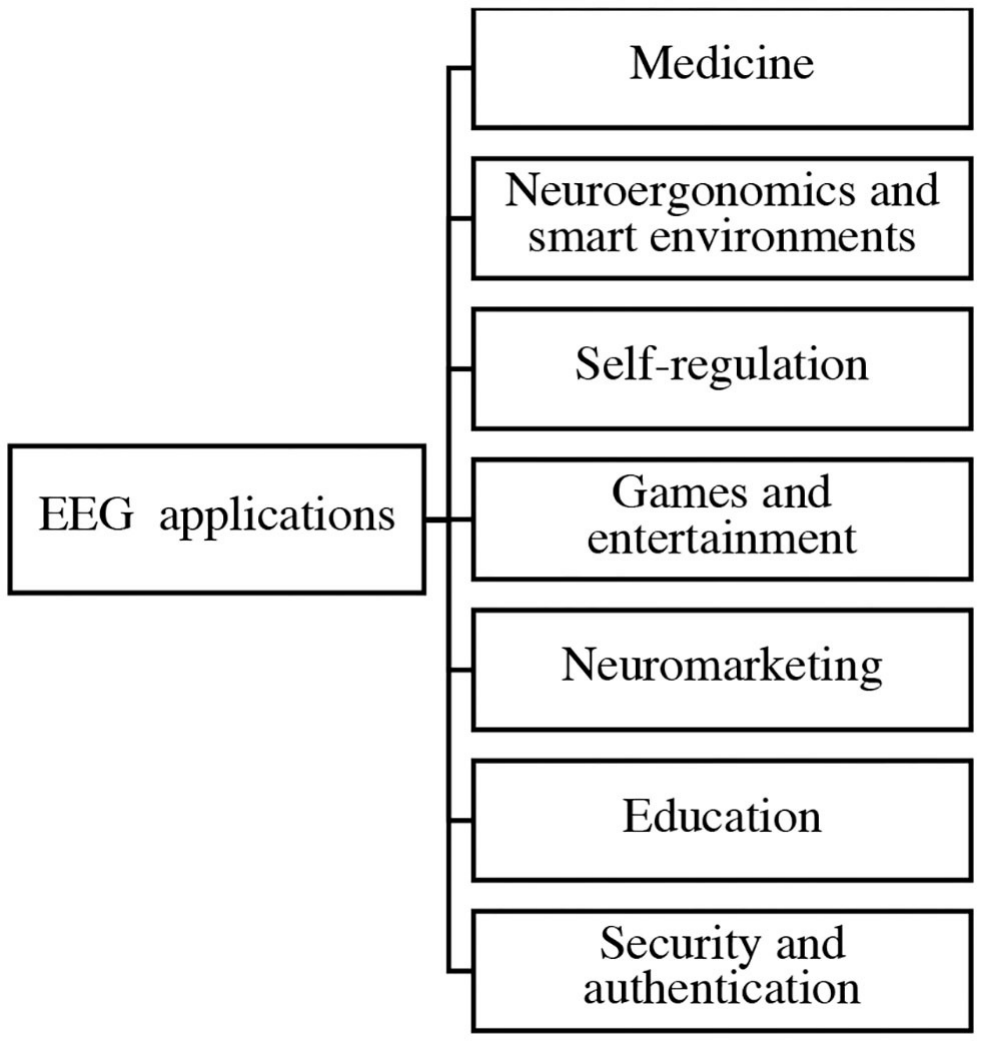
Commercial EEG potential applications in different sectors.

Following the cross-sectional review, the collected sources suggest that commercial applications of EEG are widely discussed across the literature. The most frequently cited applications include medical applications (*N* = 74), neuroergonomics and smart environment (*N* = 41), self-regulation (*N* = 26), games and entertainment (*N* = 25), neuromarketing (*N* = 21), education (*N* = 20), and security and authentication (*N* = 20). Thus, the most recent practical attempts at EEG applications are presented in Figure 2. The same depicted order will be observed in this section.

### Medicine

The most prevalent applications of EEG technologies can be found in the medical sector (*N* = 74). As identified in the literature, they are predominantly used for prediction and diagnosis of diverse clinical conditions as well as for treatment, rehabilitation and assistance of patients with certain disabilities.

#### Prediction and Diagnosis

**Figure 3:**
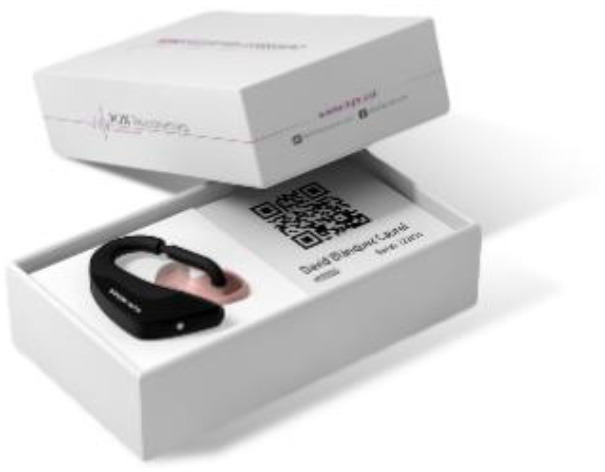
MJN-SERAS earphone EEG device for seizure prediction. Reprinted from International Epilepsy Day: Latest medical devices for epilepsy patients, by NS Medical Devices (2018), https://www.nsmedicaldevices.com/news/international-epilepsy-day-devices-epilepsy/attachment/mjn-seras-epilepsia/. Copyright 2018, by MJN Neuroserveis.

One of the main uses of EEG is for risk prediction models, which are becoming progressively more widespread for clinical aid decision-making. These models are developed to estimate the probability of having certain diseases, events or complications given the individual's demographics, test results, or disease characteristics. In this sense, EEG data can be processed for the prediction of several health problems such as sleep disorders (Kupfer et al., 1978), seizure disorders (Mormann et al., 2000; García Bellón and Soria Bretones, 2013; Sharmila and Mahalakshmi, 2017), attention deficit hyperactivity disorders (Clarke et al., 2011; Gola et al., 2013), peripheral neuropathies, and musculoskeletal diseases (Wei et al., 2010). In recent years, different commercial initiatives have been developed in the prediction of clinical conditions. One of the most ground-breaking projects for seizure prediction has been advanced by the Spanish company MJN Neuroserveis. They have developed a discreet, portable earphone device (Figure 3) capable of alerting the person and the caregiver when there is a greater risk of seizure, thus preventing falls or injury (Rincón, 2017). The start-up closed a 750,000 Euro investment round for its epilepsy prediction device in 2017, financed by investors from IESE Business School and ENISA network (Hinchliffe, 2018).

**Figure 4:**
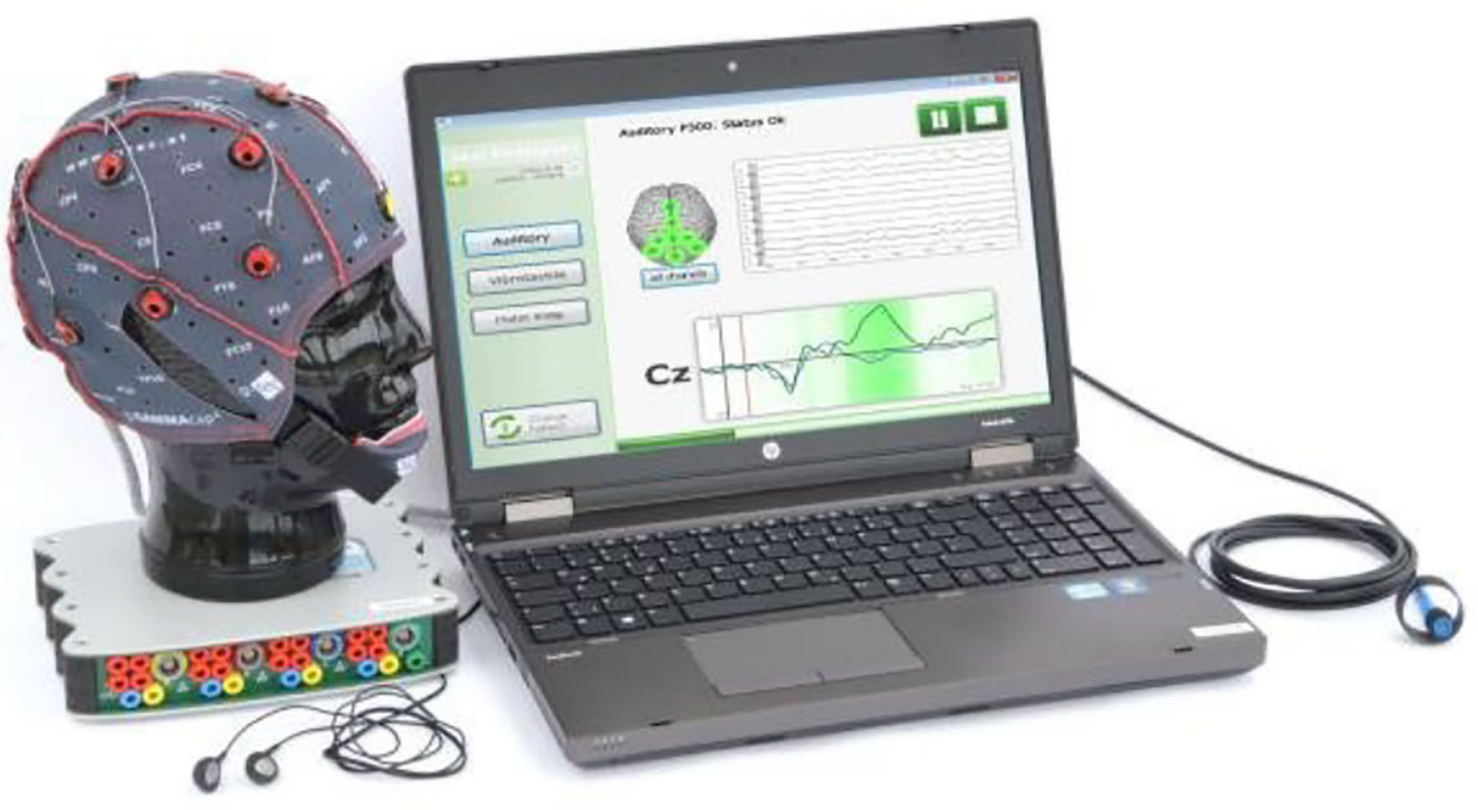
mindBEAGLE system. Reprinted from mindBEAGLE Technical Specs and Features, by g.tec medical engineering GmbH (2018), https://www.mindbeagle.at/Technical-Specs-and-Features. Copyright 2018, by g.tec medical engineering GmbH.

Moreover, EEG is also used as an initial diagnosis assessment tool alternative to MRI and CT-SCAN due to its cost-effectiveness. Here, EEG systems have proven valuable in the diagnosis of disorders of consciousness (DOC) (Guger et al., 2018; Stefan et al., 2018) as well as in the detection of tumors and concussions (Selvam and Shenbagadevi, 2011; Sharanreddy and Kulkarni, 2013; Abdulkader et al., 2015). For DOC diagnosis purposes, the Austrian company Guger Technologies (g.tec) has developed the EEG-based system mindBEAGLE (Figure 4), which provides quick and easy assessments of DOC and basic communication with certain patients (Spataro et al., 2018).

**Figure 5:**
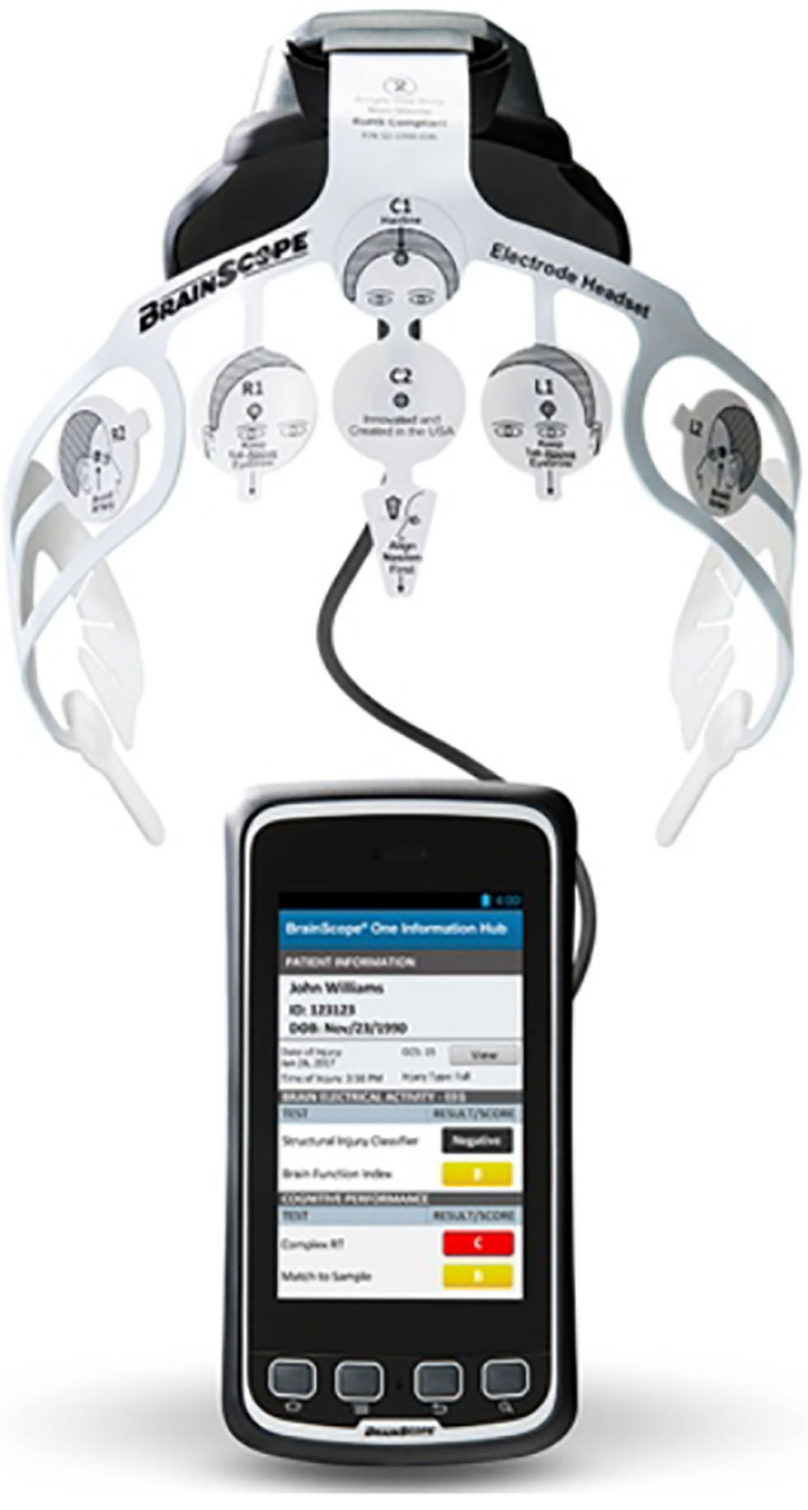
BrainScope TBI assessment system. Reprinted from Our Solutions Brainscope, by Brainscope (2018), https://www.brainscope.com/products. Copyright 2018, by Brainscope.

Also, companies such as BrainScope take advantage of EEG capabilities in order to pioneer the future of traumatic brain injury (TBI) assessment (Figure 5) (Hanley et al., 2017). At the time this review is being conducted, the company cannot actually diagnose if a tumor is present or not, so it is temporarily offering inexpensive solutions that provide preliminary insights to determine the need or not to perform a PET/MRI scan. Notwithstanding, it has been awarded more than $27 million from U.S. Department of Defense to develop its TBI and concussion assessment technology (Pai, 2015).

**Figure 6:**
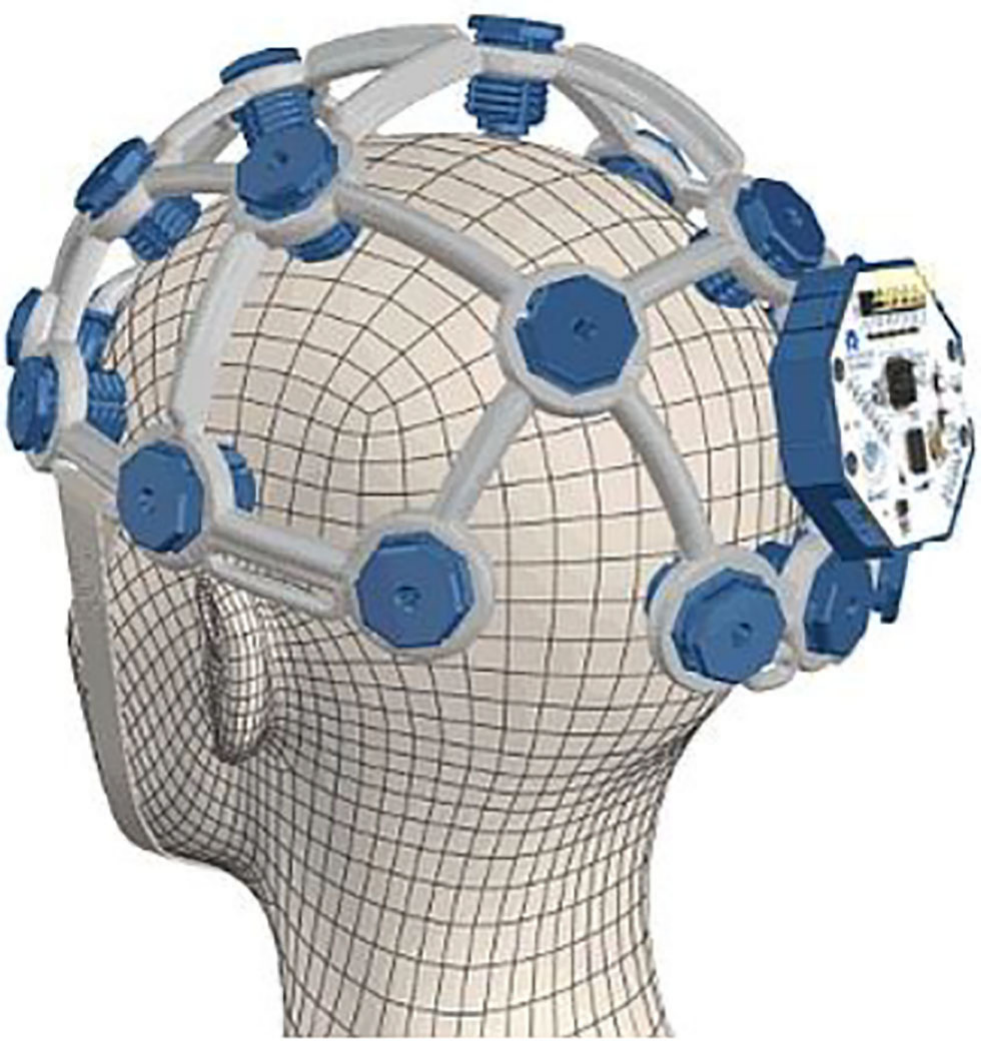
Synapto's Alzheimer diagnosis system using OpenBCI's All-in-One Biosensing R&D Bundle. Reprinted from 3D-printed brain-sensing headset is open source, by Smart2.0 (2015), https://www.smart2zero.com/news/3d-printed-brain-sensing-headset-open-source. Copyright 2017, by European Business Press SA.

In another area, EEG technologies can also be employed to diagnose neurodegenerative disorders. A growing body of evidence supports its application for early detection of Alzheimer's disease (Jaeseung, 2004; Lazar, 2018), Parkinson's disease (Solís-Vivanco et al., 2018) and for diagnosis of different dementia subtypes (Houmani et al., 2018; Stylianou et al., 2018). These studies propel the growing interest of EEG, whose market is expected to grow worldwide as geriatric population continues to increase (Nations U, 2017). Thus, new entrepreneurial initiatives sustain its attractiveness as a technology for investment. For instance, Synapto, an early stage medical technology venture founded by former students of the University of Maryland, uses portable EEG of OpenBCI to make Alzheimer's diagnosis more accessible and affordable (Figure 6). Synapto solutions are funded and validated by NIH (National Institutes of Health, 2017).

#### Treatment, Rehabilitation, and Assistance

Beyond its functions as a prediction and diagnosis tool, EEG has spread as a biomarker for treatment of several clinical conditions. In particular, EEG biofeedback can be used for the treatment of patients suffering from addictions due to its direct correlation with drug dependency (Prichep et al., 1996; Trudeau, 2005). Furthermore, it can also be applied as a potential therapy to help patients with Rett syndrome (Fabio et al., 2016) and for memory deficits recovery via neurofeedback (Rozelle and Budzynski, 1995; Kober et al., 2015). Although EEG does not provide complete relief in all cases, it can assist patients with managing their symptoms, thus affording them a better life quality.

**Figure 7:**
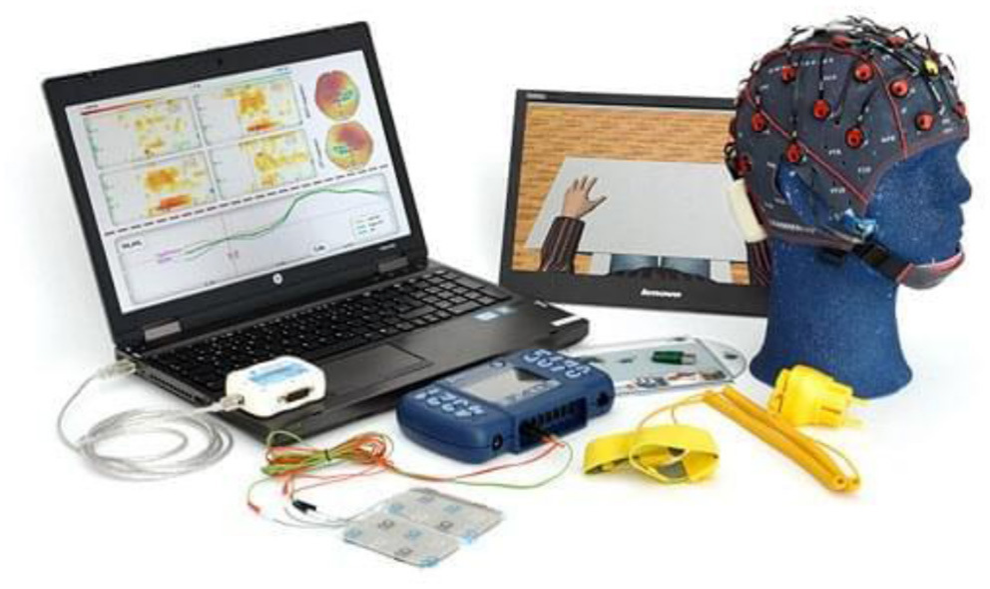
RecoveriX rehabilitation system. Reprinted from RecoveriX system, by Madisson Medical and Welness Technology (2018), https://www.madisson.cz/en/product/recoverix-system. Copyright 2018, by g.tec medical engineering GmbH.

On the other side, despite the superior capabilities of invasive methods, the viability of EEG for rehabilitation and restoration of lost functions cannot be ignored. In the light of overwhelming number of scientific evidence, EEG may be a feasible tool for the treatment of in lock-in syndrome (LIS) patients and patients with severe motor disabilities (Markand, 1976; Birbaumer and Cohen, 2007; Sellers et al., 2010; Zickler et al., 2011). It may be also a valuable bedside tool for neuro-motor rehabilitation on post-stroke patients (Markand, 1976; Birbaumer and Cohen, 2007; Ang et al., 2010; Sellers et al., 2010; Zickler et al., 2011; Comani et al., 2015). Here, EEG can be applied to regain previous levels of mobility or, at least, it can allow patients to better manage their dysfunctionalities. As stroke rehabilitation is a very active direction in this field, many products are being deployed for commercial purposes such as recoveriX (Figure 7), a g.tec solution which is currently being franchised in different treatment centers around the world, or nBETTER, a system created by the Singaporean company Neurostyle Pte Ltd. which detects visualized movements of stroke-affected limbs using EEG-based neuro-feedback to provide visually engaging and mechanical feedback.

**Figure 8:**
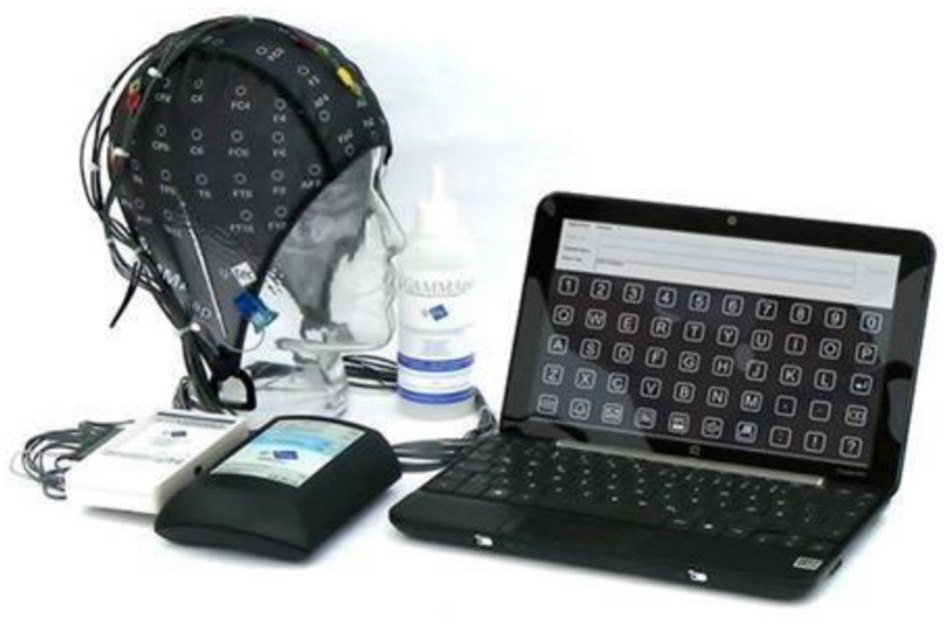
IntendiX spelling system. Reprinted from Intendix, The Brain Computer Interface Goes Commercial, by Singularity Hub (2010), https://singularityhub.com/2010/03/07/intendix-the-brain-computer-interface-goes-commercial-video/. Copyright 2010, by g.tec medical engineering GmbH.

As for assistive purposes, EEG permits disabled people to communicate their opinions and ideas via a variety of methods such as spelling applications (Birbaumer and Cohen, 2007; Akcakaya et al., 2014; Birbaumer et al., 2014; Rezeika et al., 2018), semantic categorization (Stothart et al., 2017), or silent speech communication (Brumberg et al., 2010; Mohanchandra et al., 2015). This may facilitate advanced hands-free applications, which may provide disabled people ease and comfort. In this realm, the world's first personal EEG-based spelling system was introduced in 2010 by g.tec (Fazel-Rezai et al., 2012). Besides writing a text, the user can also interact with the system (IntendiX) to trigger an alarm, print out or copy texts into an e-mail, or send commands to external devices (Figure 8).

**Figure 9:**
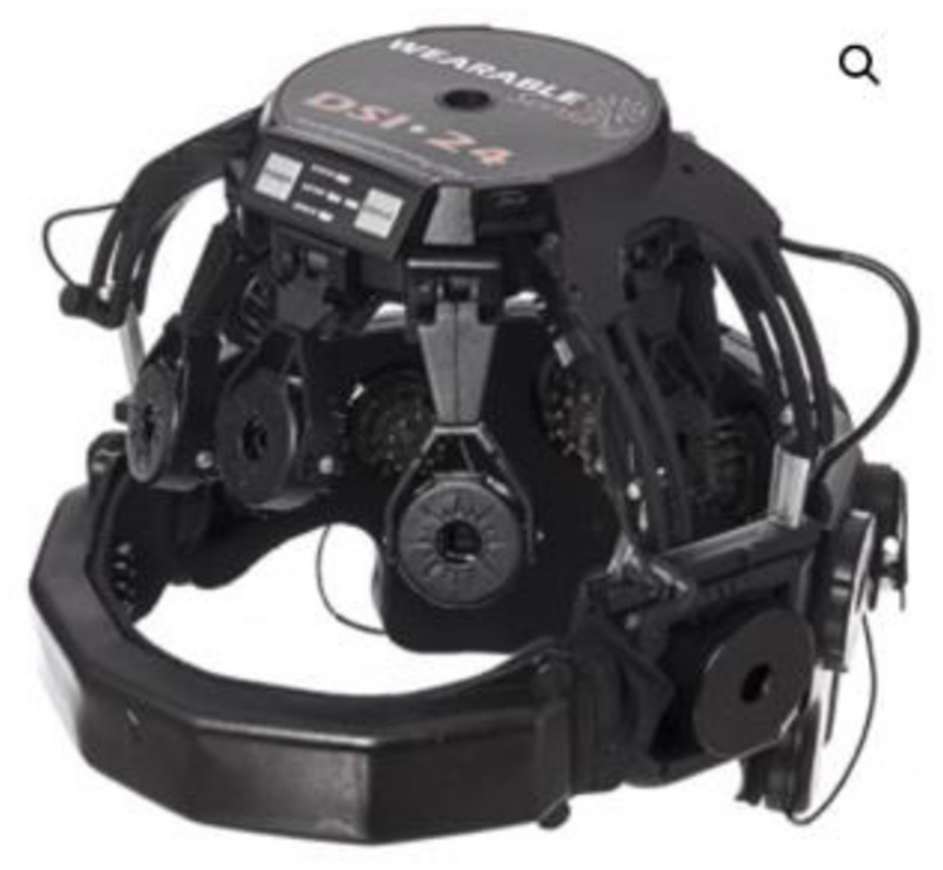
Neuracle's brain-controlled keyboard using DSI24 headset. Reprinted from DSI 24 Dry Electrode EEG Headse, by Wearable Sensing (2018), https://wearablesensing.com/products/dsi-24/. Copyright 2018, by Wearable Sensing.

Likewise, Neuracle -a spinoff startup of Tsinghua University- developed a high-speed brain-controlled keyboards using WearableSensing's DSI24 headset (Figure 9), which has achieved high spelling rates up to 60 characters (~12 words) per minute (Chen et al., 2015).

Other companies offering diverse EEG solutions for medical applications are listed below (Table 1). The list ranks several hardware companies according to their number of publications, as found from Google Scholar. While it is not an exhaustive list, it represents an overview of some of the most important key players.

**Table 1:** EEG key-players.

**Company**	**Number of publications**	**Main product**
NeuroScan	15.200	“Quick Caps” headsets up to 256 electrodes
BioSemi	8.800	“HeadCap” headset up to 256 channels
Brain products	6.690	“BrainCap” headsets up to 160 channels
g.tec	6.260	“g.Nautilus” headset 64 channels
EGI	5.000	“Geodesic EEG System 400” up to 256 sensors

### Neuroergonomics and Smart Environments

The use of EEG is also widely extended (*N* = 41) for the design of safer and more efficient operational environments in relation to neuroscience principles (Karwowski et al., 2003). This area of research has been called neuroergonomics (Parasuraman, 2003) and it is applicable to different contexts of usage.

One of the most attractive applications of neuroergonomics are smart environments. As a consequence of the progress in sensors and information technology, it has been identified that the feasibility of these environments is being gradually consolidated (Kameas and Calemis, 2010; Kosmyna et al., 2016). This new paradigm may enable human interaction with digital environments that are sensitive, adaptive, and responsive. In this line, several authors have reported that BCI assistive technologies related to automation and control of ubiquitous devices may have a promising impact on such intelligent settings (Pfurtscheller et al., 2006; Navarro et al., 2011). The next generation of human-compatible systems, powered by EEG-based BCI and ubiquitous computing, may not only help despaired people to regain higher standards of autonomy but may also drive the expansion of the living conditions of people, thus ensuring great comfort along with the intelligent usage of resources (Domingo, 2012; Lee et al., 2013; Corralejo et al., 2014; Kosmyna et al., 2016). Although some authors have reported the difficulty of building EEG systems for smart environments given the current state-of-the-art (Aloise et al., 2011; Su et al., 2011; Mehta and Parasuraman, 2013), significant initiatives can be found in this field. For instance, BrainAble, an ongoing European Seventh Framework Program (FP7) financed project, aims to develop a multimodal neuronal interface with affective computing and virtual environments to restore and improve functional independence of patients with motor disabilities in their activities of daily living. The project also attempts to connect a human-computer interface with adapted social networks services in order to improve the life quality of the patient (Carmichael and Carmichael, 2014).

**Figure 10:**
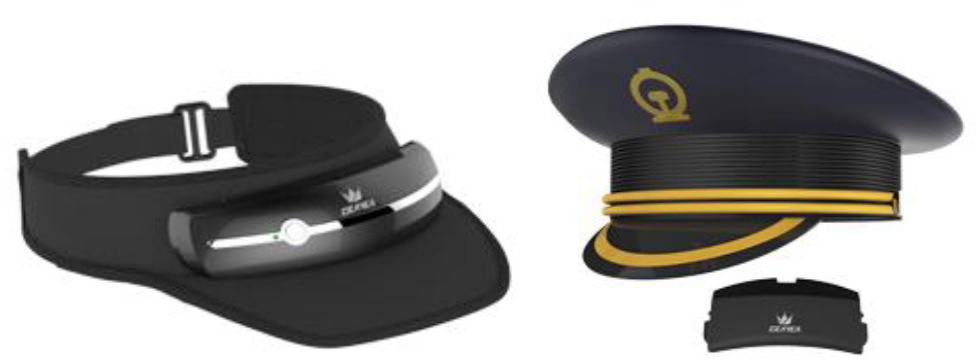
Deayea's driver headsets. Reprinted from Smart headset, by Shanghai Diyi Technology Co., Ltd. (2018), http://www.deayea.cn/page98?product_id=4. Copyright 2018, by Shanghai Diyi Technology Co., Ltd.

Neuroergonomic principles can be also employed in workplace environments. Here, neuroergonomics focuses on designing and controlling physical tasks to ensure that work demands are adapted to the physical, cognitive, and affective capabilities and limitations of the operator (Venthur et al., 2010; Garcia-Molina et al., 2013). Thus, EEG systems can be potentially used for cognitive real-time monitoring of workers mental workload in order to alert them to trigger certain behaviors. In the automotive sector, several studies have investigated the use of EEG during driving simulations for assessing driving performance and inattentiveness, and for detecting needs of emergency brakes before the braking onset (Dong et al., 2011; Karthaus et al., 2018). By doing so, authors have identified that distraction and fatigue are two main sources for driver's inattention, which in turn is considered as a strong cause for most traffic accidents (Dong et al., 2011). Those insights may be useful for technology transfer purposes, which may eventually propel the emergence of new companies as a result of the new advances in neuroergonomics. Deayea, a Chinese Shanghai-based company, is reportedly using EEG sensors in the caps of train drivers on the high-speed rail line between Beijing and Shanghai to monitor their concentrations levels and to identify thoughts of anger, anxiety, and sadness (Figure 10) (Chan, 2018). In the aviation sector, initial steps have also been taken toward assistive technologies for prevention of accidents, particularly in air traffic control and aircraft piloting (Fricke et al., 2014; Aricò et al., 2016; Vecchiato et al., 2016). By expanding these technologies, a new generation of wearables that enhance human performance or fully adapt user interfaces to different environments may be achieved. These wearables could be ultimately used as a tool to prevent the risk of error in operational environments.

Along the same line, advances in neuroergonomics may not only foster EEG solutions in workplace environments but also in the vast consumer markets. As EEG enables the identification of attention levels while doing a certain task, the future of driving could be disrupted. For instance, Nissan is developing a way to help drivers execute evasive maneuvers faster by using EEG technology. The project, called Brain-to-Vehicle, attempts to recognize the mental and emotional states of the driver in order to help semi-autonomous cars begin evasive actions between 0.2 and 0.5 s faster. Although it is at the moment in the experimental phase, it aims to develop practical applications within 5 to 10 years (O'Kane, 2018).

### Self-Regulation

A growing body of evidence (*N* = 26) indicates that self-regulation through tracking devices plays an important role in the voluntary control of mental and physiological processes (Lomas et al., 2015; Tang et al., 2015). In this manner, self-regulation through EEG has been claimed to be beneficial for wellbeing and emotional balance, especially in mindfulness meditation (Lutz et al., 2006, 2008; Rodina et al., 2017). Thanks to its efficacy as a brain activity tracking tool device, EEG is experiencing a steady growth in these kinds of market applications. Its most paradigmatic uses are being developed for meditation, focus, and sleep purposes.

**Figure 11:**
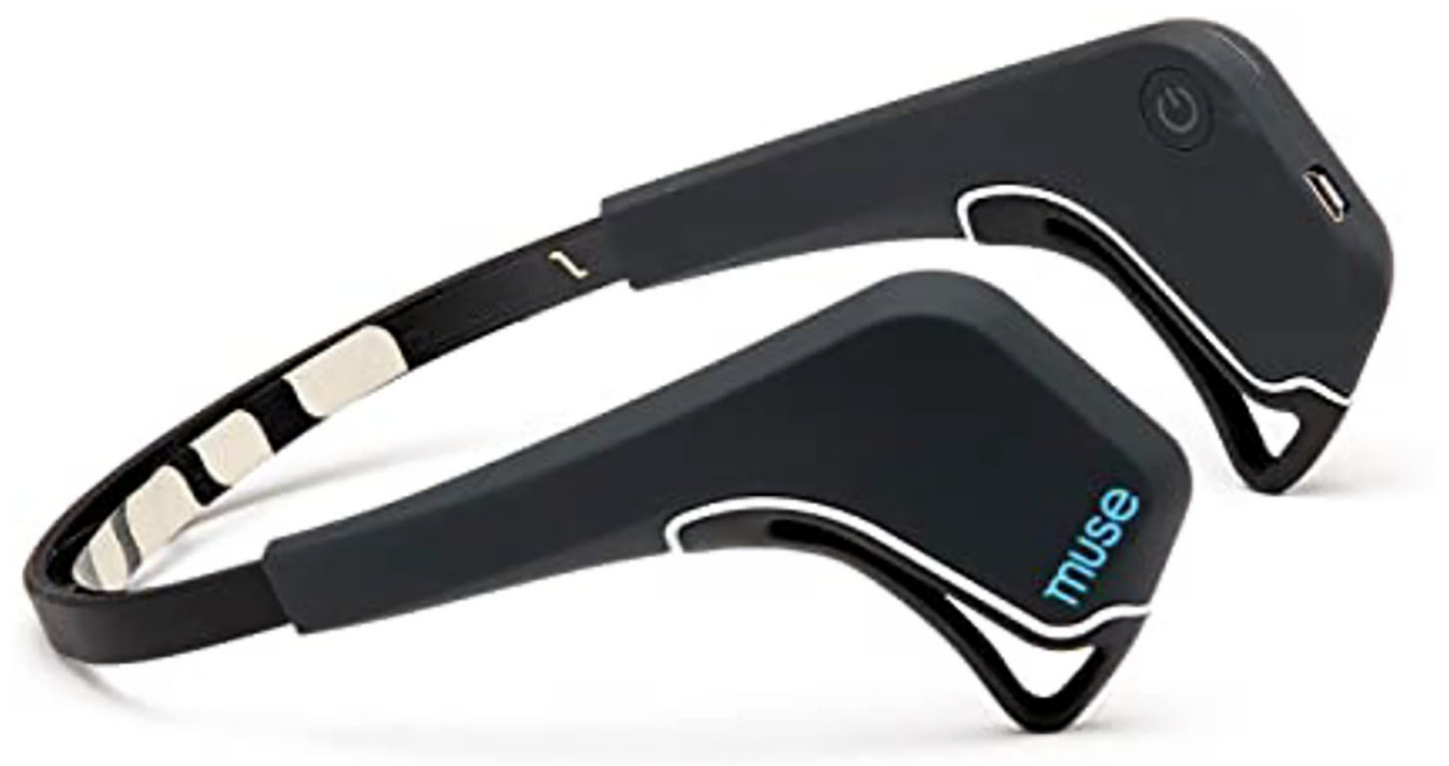
Muse's headband. Reprinted from Muse: the brain sensing headband, by Amazon (2018), https://www.amazon.com/Muse-Brain-Sensing-Headband-Black/dp/B00LOQR37C. Copyright 2018, by Muse.

In the meditation sector, the Canadian company InteraXon launched in 2014 the wearable headset Muse (Li et al., 2015), which measures user's brain activity and converts the EEG signal into audio feedback that is fed to the user via headphones, thus guiding the user during the whole meditation process (Figure 11). Muse also tracks users' progress and sets goals to keep them motivated.

**Figure 12:**
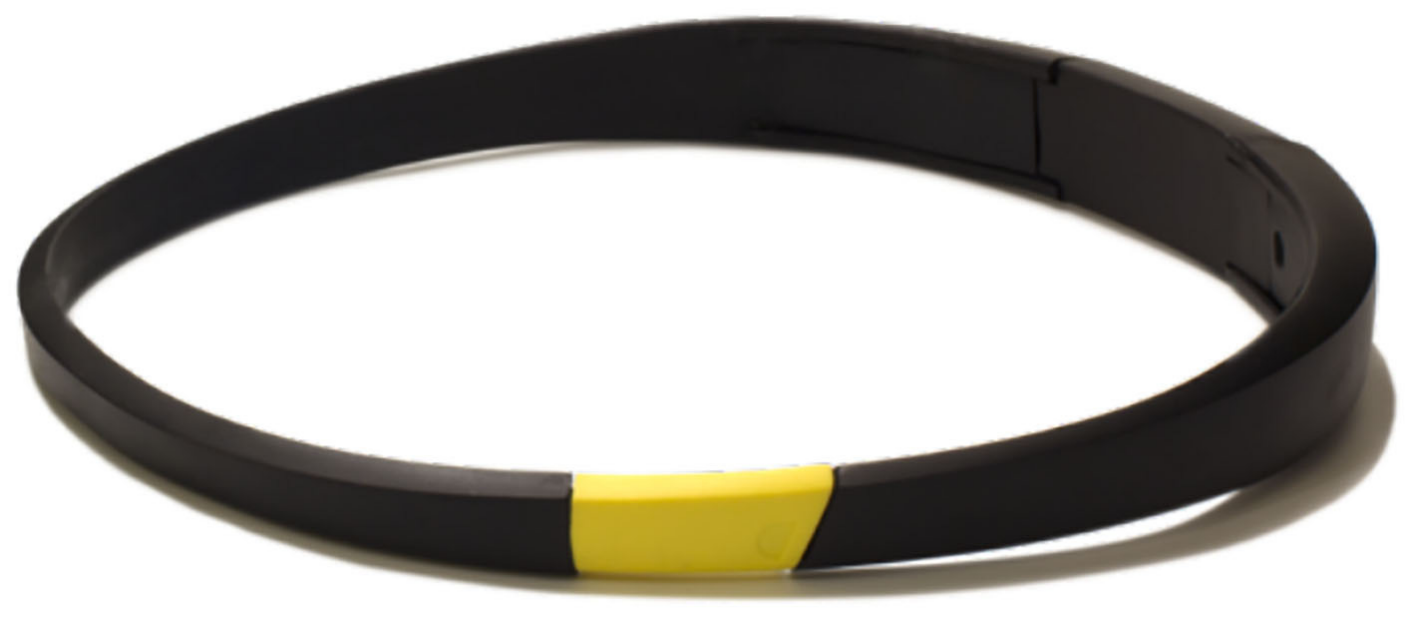
Melon's headband. Reprinted from Gadgets to help you relax in the new year, by ZDNET (2016), https://www.zdnet.com/pictures/8-gadgets-to-help-you-relax-in-the-new-year/5/. Copyright 2016, by Melon.

Further, for focus purposes, the company Melon, which was crowdfunded in 2011 in Kickstarter, has developed an algorithm which identifies the attention levels of the user in relation to its activities and behaviors (Melon, 2016). By doing so, they aim to develop a neural tracking tool solution to improve user's productivity (Figure 12).

**Figure 13:**
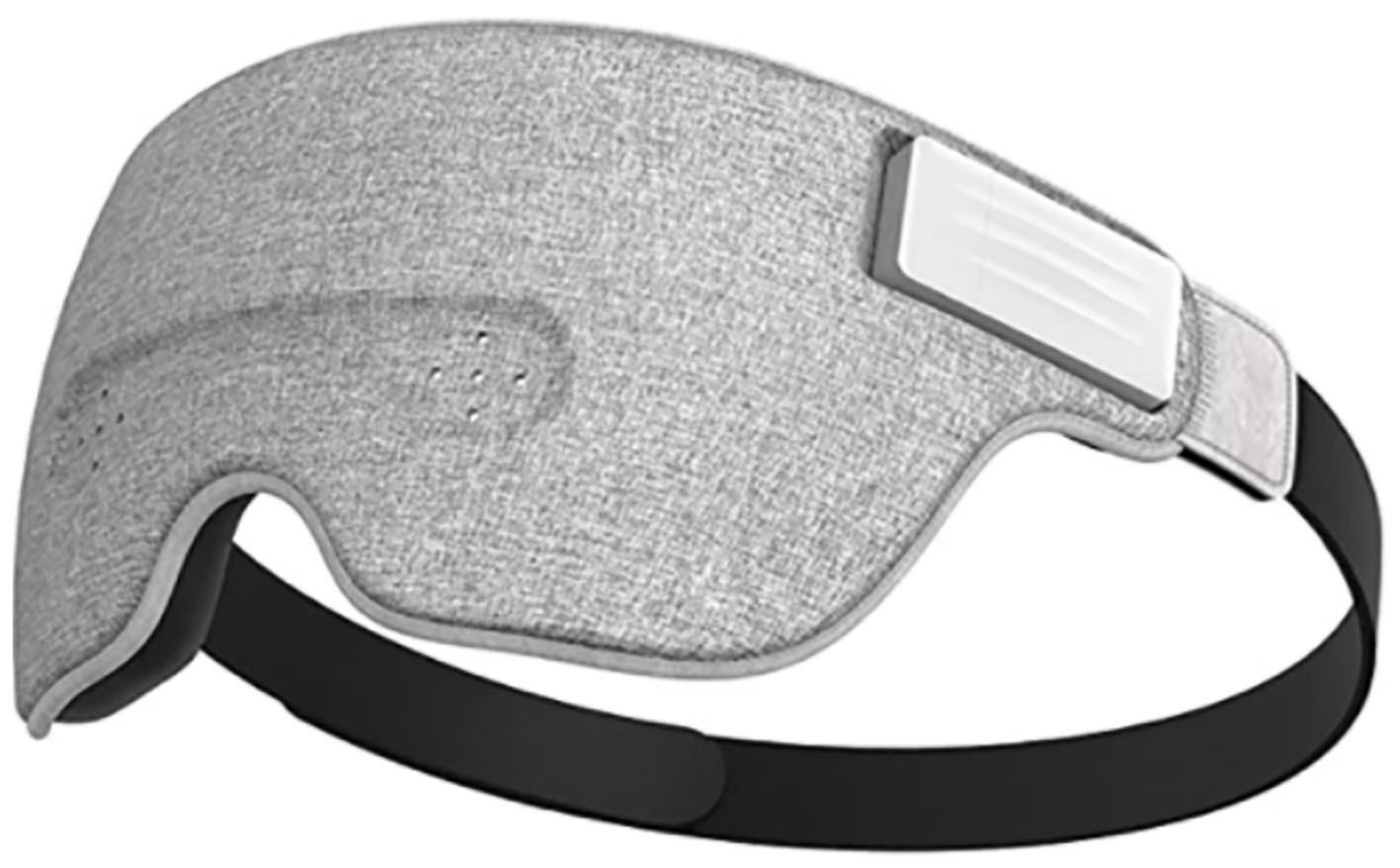
Lunna headset. Reprinted from Ivation Luuna Brainwave Brain Sensing Bluetooth Smart Sleep Mask, by Amazon (2018), https://www.amazon.com/Ivation-Brainwave-Bluetooth-Connection-Technology/dp/B07RYNBTJW. Copyright 2018, by Entertech.

In the sleep sector, smart sleeping masks have been developed to improve the sleeping habits of consumers. Companies such as Entertech and Neuroon use these masks to track neural activity while napping (Figure 13). For instance, Entertech's mask Lunna recognizes rhythmic activities in the alpha range during the drowsiness period at sleep onset and in the rapid eye movement (REM) sleep stage in order to wake users up in the light sleep, thus preventing post naps to cloud the user's day (Lunna, 2018).

### Games and Entertainment

**Figure 14:**
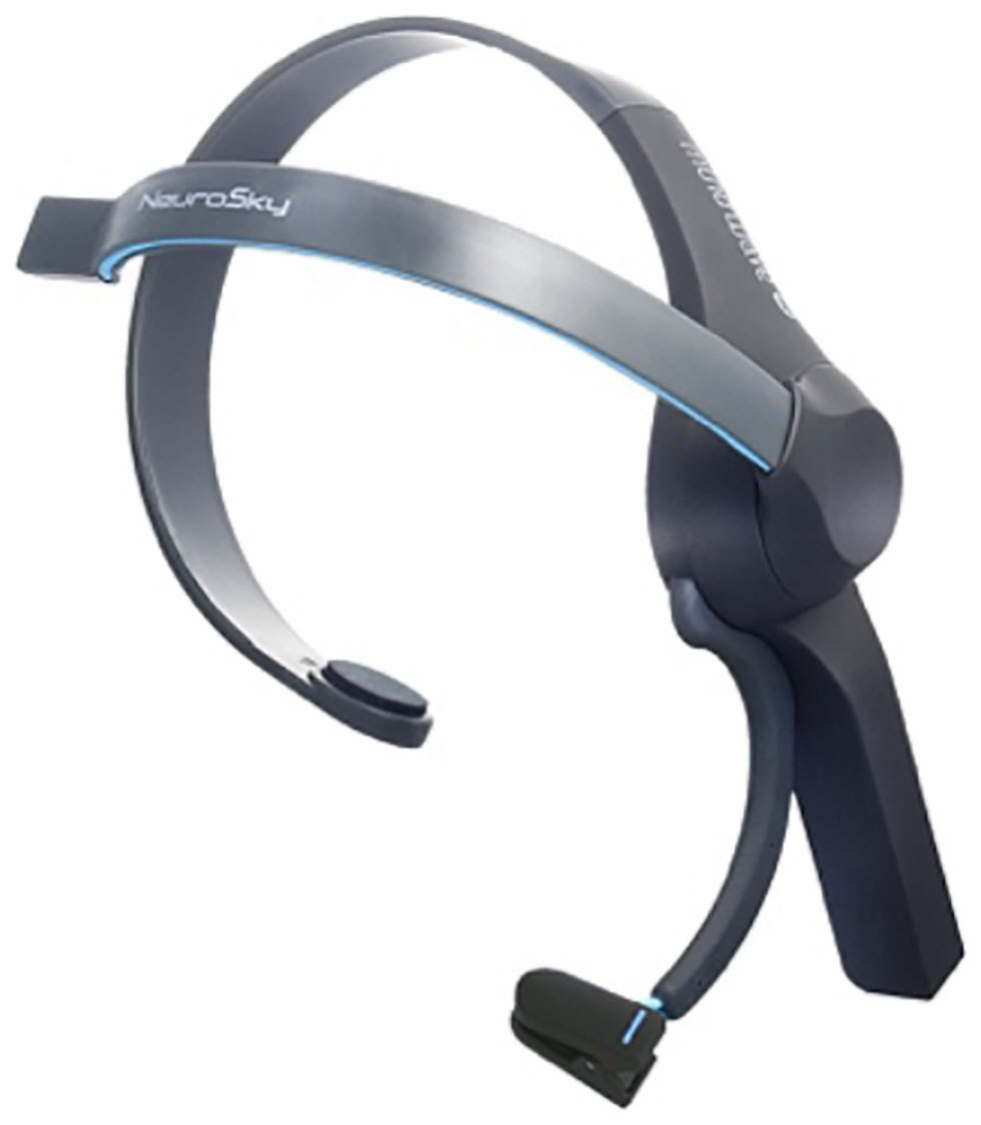
MindWave headset. Reprinted from NeuroSky MindWave Mobile BrainWave Starter Kit, by Amazon (2018), https://www.amazon.in/NeuroSky-MindWave-Mobile-BrainWave-Starter/dp/B00B8BF4EM. Copyright 2015, by NeuroSky.

Games and entertainment are industries which will benefit extraordinarily from the deployment of portable and ergonomic EEG. In the academic sphere, the literature shows that most games and entertainment applications focus on multi-dimensional control using motor imagery-based EEG systems in virtual environments or in three-dimensional physical space (*N* = 25). In virtual environment games, most research has concentrated on classification performances and user experience (Doud et al., 2011; Bonnet et al., 2013). Hence, Bonnet et al. created a multi-user game called BrainArena in which two users play football by means of EEG-based BCI (Bonnet et al., 2013). In three-dimensional physical space, LaFleur et al. presented a novel experiment of EEG-based BCI controlling a robotic quadcopter, which reported high control from remote distances with fast and accurate actuation (LaFleur and Nemec, 2013). In the commercial sphere, American companies such as NeuroSky and Emotiv are leading the industry of games and entertainment. NeuroSky's headset MindWave works with many gaming apps in the NeuroSky store, an in-house digital distribution platform which offers a wide variety of brain-controlled apps (Figure 14). One of the most popular games in this platform is BrainCopter, a game that allows users to command a virtual helicopter which should evade oncoming enemies by means of MindWave headset.

**Figure 15:**
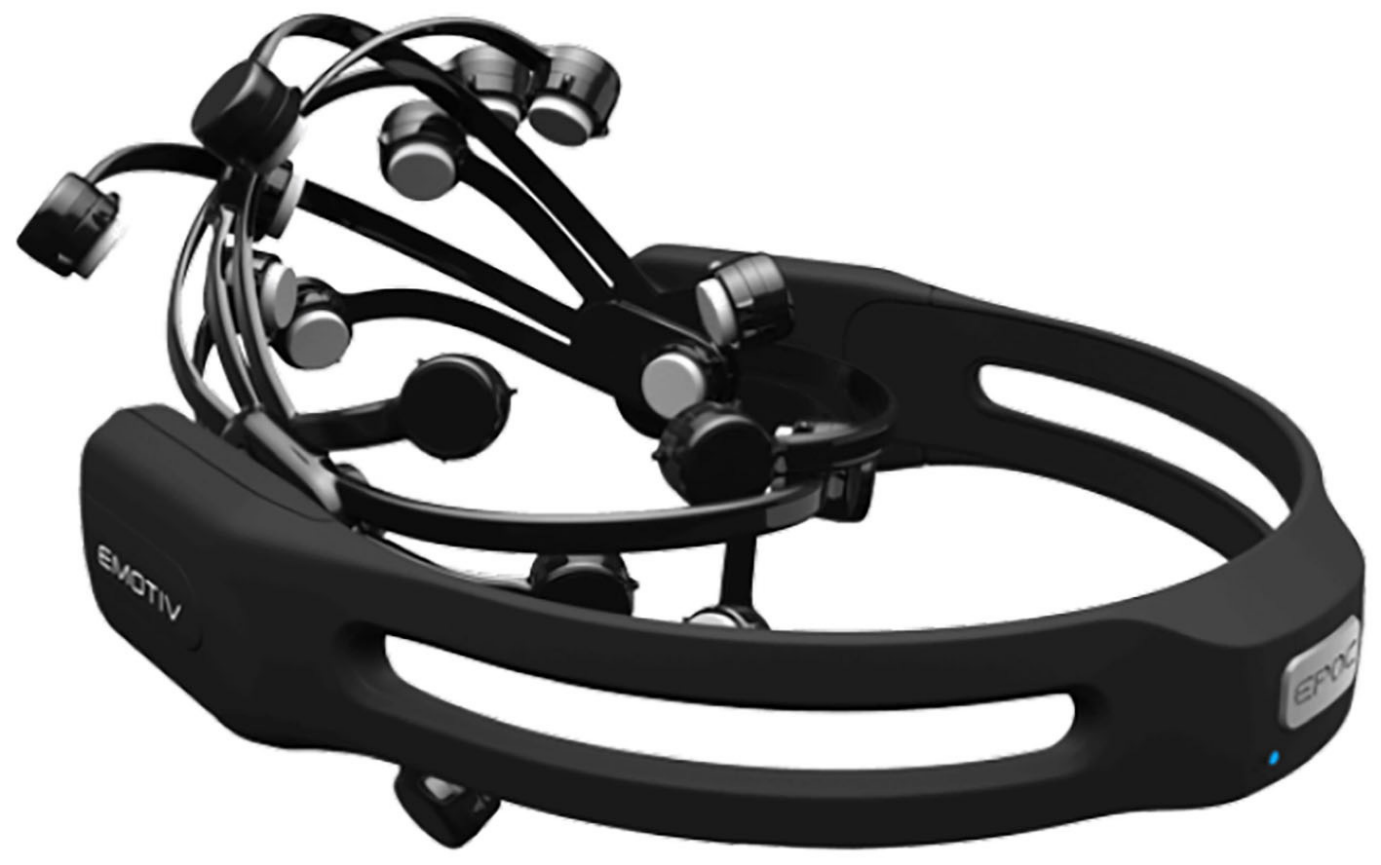
Emotiv EPOC+. Reprinted from Emotiv EPOC+, by EMOTIV (2018), https://www.emotiv.com/epoc/. Copyright 2018, by EMOTIV.

In physical space environments, Emotiv's EPOC+ and Insight headsets similarly enable users to control drones remotely (Figure 15) (Wang et al., 2018).

Other efforts done in this area focus on making virtual reality environments and video games more immersive by using EEG as a control input (Lécuyer et al., 2008). In general, experiments conducted in this realm combine the use of different wearables. On the one hand, the user benefits from a virtual reality (VR) video game experience via VR headsets such as Oculus Rift (Doma, 2018). On the other hand, an EEG headset monitors the player's brain activities and digitizes it as a computer input for the VR video game.

**Figure 16:**
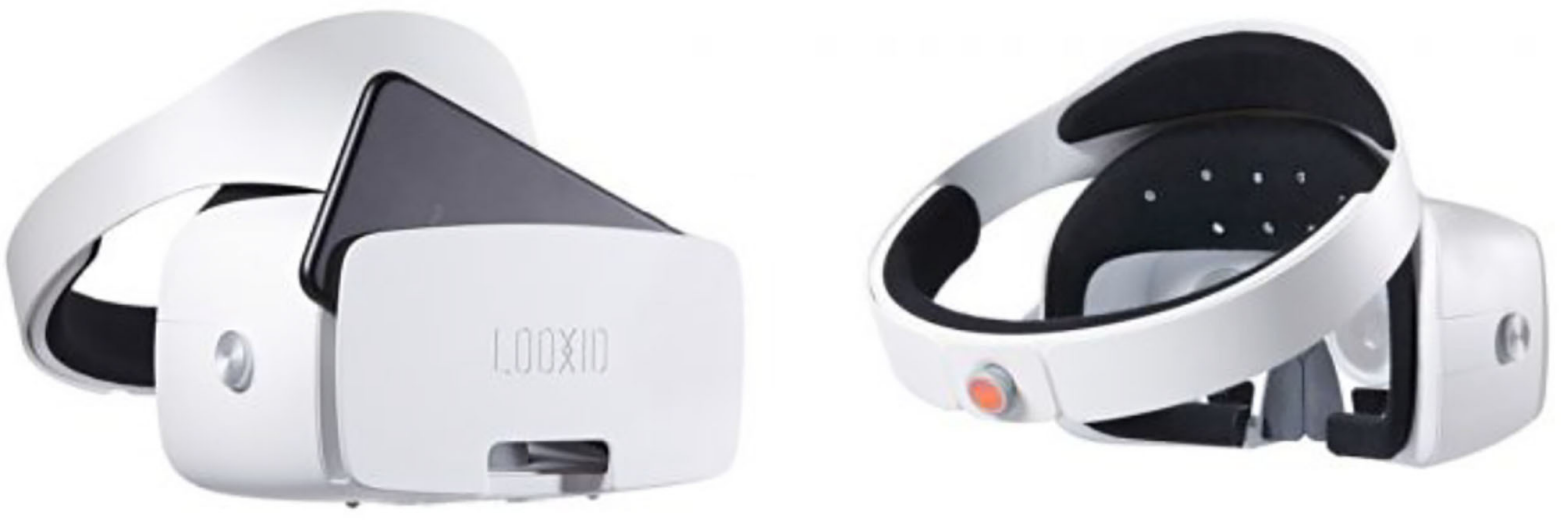
LooxidLink headset. Reprinted from Looxid Labs, by Seamless (2018), https://shiropen.com/2017/09/19/28240/. Copyright 2018, by Seamless.

Considering the integrative potential of both technologies, manufacturers are starting to incorporate eye-tracking and EEG sensors in the VR headsets to allow brain-controlled portable solutions. One of the most innovative products on the market is the mobile-powered VR headset LooxidLink (Figure 16). This wearable, designed by the Korean company Looxid Labs, integrates gold-plated EEG sensor capabilities into the VR components of traditional VR headsets, thus allowing the user to take advantage of the commands in the API to apply EEG into the VR environment. Looxid Labs won the Best of Innovation Award at Consumer Electronic Show 2018 (Jo, 2018).

### Neuromarketing and Advertisement

Neuromarketing is an emerging interdisciplinary field located at the borderline between neuroscience psychology and marketing. The term neuromarketing was initially introduced by Ale Smidts in 2002 and is defined as “the study of the cerebral mechanism to understand the consumer's behavior in order to improve the marketing strategies” (Boricean, 2009; Stasi et al., 2018). Neuromarketing focuses on assessing consumers' cognitive and emotional responses to various marketing stimuli (Karmarkar, 2011). To achieve this purpose, neuromarketing studies can be conducted by means of different non-invasive techniques such as fMRI, MEG or PET.

The use of EEG has only recently been adopted in confluence with implicit associations' test and other biometric techniques, such as eye-tracking, psychophysiological and electrodermal reactivity, or heart and respiratory rate (Calvert and Thensen, 2004; Kenning and Linzmajer, 2011; Morin, 2011). By means of EEG, effectiveness indicators such as emotional engagement, memory retention, awareness, and attention can be measured (Vecchiato et al., 2011; Sebastian, 2014). For instance, EEG evaluation has demonstrated remarkably results for TV commercials, where attention levels have been successfully measured, thus providing researchers with new methods for advertisement evaluation (Vecchiato et al., 2009; Nomura and Mitsukura, 2015; Wang et al., 2016). Several authors have reported that the analysis of these indicators is fundamental to discovering the factors that influence consumers' purchase decisions (McClure et al., 2004). Also, they may contribute to the better understanding of consumers' thoughts, emotions, feelings, needs, and motivations, as related to the purchasing process (Lindstrom and Underhill, 2010).

Although EEG is relatively a new consumer neuroscience technique, its application has hastily grown over the past years (*N* = 21) (Plassmann et al., 2012; Smidts et al., 2014). Some of the most important milestones for EEG in the commercial arena include Yahoo's assessment of consumers' reactions to television commercials; Hyundai's measurement of consumer neurological responses when viewing a sports car prototype; and Microsoft's assessment of the degree of consumer engagement when using an Xbox video game (Flores et al., 2014). According to Plassmann et al., more than 300 companies are currently working worldwide in the field of neuromarketing (Plassmann et al., 2012). Among the vendors of these neuromarketing services are three American-based neuromarketing companies: NeuroFocus, a company absorbed by Nielsen Holdings, working with Hyundai, Google and Walt Disney Co.; EmSense, which counts Microsoft among its customers; and Sands Research, which collaborates with Chevron (Flores et al., 2014).

### Education

In the educational sector, EEG is mainly used to track student performance to improve the learning experience (*N* = 20).

During the learning process, student's attention and motivation during instruction generally influence the understanding of the contents (Saeed and Zyngier, 2012; Ning-Han et al., 2013). However, traditional teaching methods require teachers to visually detect students' expressions in order to infer whether they are thoughtfully learning or not. Of course, this method poses a physical burden to the teachers and is not always infallible.

By applying neural technologies to provide instant feedback on the mental levels of students, the shortcomings of traditional teaching methods may be remedied. Several studies have corroborated this approach. For example, the feasibility of collecting useful information about cognitive processing and mental state using portable EEG monitoring devices has been already assessed by Mostow et al. (2013). Also, the development of visual attention measurement systems based on EEG are under current development (Ko et al., 2017). These findings may set the basis for developing EEG systems capable of estimating the level of cognitive and visual attention during real classroom activities, thus enhancing the learning effectiveness (Corentin and Pascal, 2013).

Teachers may vastly benefit from this technology, which may alleviate their inaccuracy and reduce their burdens in measuring attention levels of learners (Slavin, 2008; Xu and Zhong, 2018).

**Figure 17:**
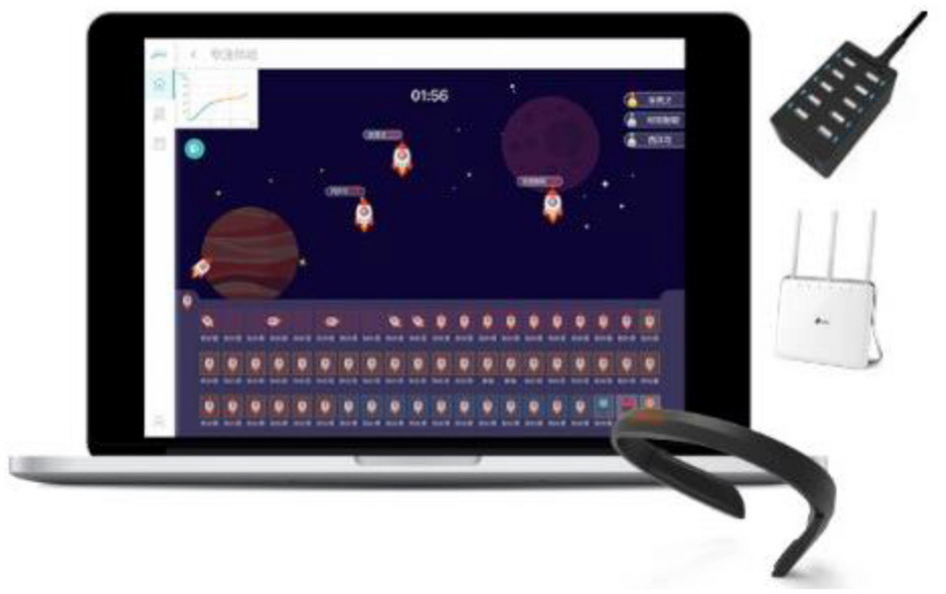
FocusEDU system. Reprinted from BrainCo Focus1 Headband And FocusEDU Software - Classroom Pack, by Tierney (2018), https://www.tierney.com/products/brainco-focus1-headband-and-focusedu-software-classroom-pack/. Copyright 2018, by Tierney Brothers, Inc.

A representative example of this methodology is Harvard Innovation Lab's incubated BrainCo. BrainCo has developed an EEG headband (FocusEDU) which aims at helping students cultivate efficient and focused habits through neurofeedback training (Figure 17).

By means of its accompanying app, which can be visualized on a tablet or a computer, FocusEDU can also be applied in the classroom to improve educators' teaching methods by tracking students' levels of engagement and attention. In 2017, BrainCo won the Most Innovative Award at the International Society for Technology in Education (ISTE) Conference (Smith, 2017).

### Security and Authentication

The use of EEG data as a biometric trait for security and authentication purposes has experienced a tremendous growth in cryptographic and biometric frameworks (*N* = 20). Although many EEG-based authentication methods have been proposed, they have been roughly divided into two categories depending on the presence or absence of a stimulus. The former comprises both eyes-open/eyes-closed, whereas the latter includes visual evoked potentials, mental tasks, and emotional stimuli (Wu et al., 2018). In general terms, the use of EEG in these fields has been propelled due to the need for data security and authentication in numerous applications such as e-commerce, e-health, e-government, e-voting, or blockchain, among others (Damaševičius et al., 2018). More precisely, in cryptographic frameworks, security systems have shown to be vulnerable to several drawbacks such as simple insecure password, shoulder surfing, theft crime, and cancelable biometrics (Khalifa et al., 2012). One of the main concerns about these vulnerabilities is the absence of connections between verification strategies and the identity of the person (Karthikeyan and Sabarigiri, 2011). Thus, unlike cryptographic based authentication methods, cognitive biometrics can remedy these obstacles as they can uniquely identify a person based upon independent physical or behavioral characteristics (Svogor and Kisasondi, 2012; Ramzan and Shidlovskiy, 2018). Another motivation behind the exploration of bio-signals is that they cannot be casually acquired by external agents and they are present in every living being, which gives them advantages over other biometric-based authentication methods such as iris, fingerprints, face, palm, voice, and gait recognition (Revett et al., 2010). In addition, several studies have reported that cognitive-based biometric systems offer more resistance to spoofing attacks due to the difficulty of synthesizing EEG signals, and they have also tested covert warning messages when authorized users are in a condition of external forcing (Su et al., 2012).

As for the accuracy of these systems, research shows that the gamma-band of visually evoked potential signals and the neural network classifier could be used to identify individuals. Here, Palaniappan (Palaniappan, 2004) identified 20 individuals with an average accuracy of 99.06% and Hema et al. (2008) reached an average accuracy of 94.4 to 97.5% on 6 subjects. EEG signals have been also used in user context environments, such as simplified driving simulators, where they have been processed to verify the driver's identity on demand (Nakanishi et al., 2011). Likewise, several types of research have considered the authentication of EEG signals generated from driving behavior as part of smart driving systems. For example, EEG signals could be processed to characterized alcoholic drivers. As indicated by Murata et al., the deployment of these systems may help to prevent fatal incidents (Murata et al., 2011).

In the commercial arena, analysts predict that the global EEG biometrics market is to expand at a compound annual growth rate of 12.37% during the period 2016–2020 (Damaševičius et al., 2018). Meanwhile, efforts have been also made to create open-source authentication communities such as NeurotechX, who is currently developing an EEG biometric authentication system called Brainlock based on N400 (Swaine-Simon, 2017).

## Ethical Aspects

**Figure 18:**
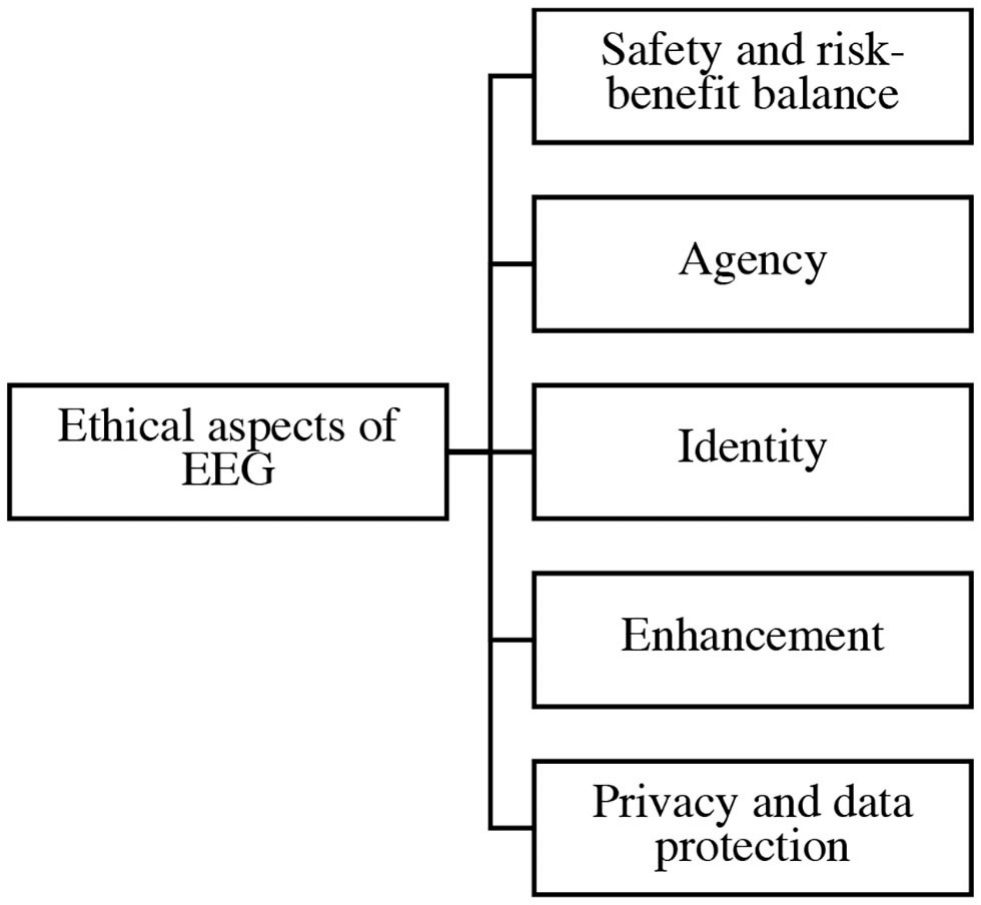
Ethical aspects of EEG.

The review collected sources revealed that ethical issues are also broadly discussed across the literature. It was found that ethical aspects may be treated whether as the subject matter of the paper or as a related subsection of an engineering study. Furthermore, it was noted that most of the articles deal with more than one ethical issue in depth and mention several other ethical aspects. The most frequently cited issues include safety and risk-benefit balance (*N* = 33), agency (*N* = 28), identity (*N* = 24), enhancement (*N* = 21), and privacy and data protection (*N* = 15) (Figure 18). Thus, the same depicted order will be observed in this section.

### Safety and Risk-Benefit Balance

Among all the concerns surveyed in the literature, the most commonly cited problems involve the safety of EEG devices and their related balance of risks and benefits (*N* = 33). These dimensions of concern are in accordance with the intrinsic hazards of any biomedical device. It was also determined that safety and risk-benefit balance mainly refer to their medical and non-medical consequences.

With regards to the medical hazards, authors assert that non-invasive devices may pose serious risks of harm (Tamburrini and Mattia, 2011). Their main concerns relate to the negative side effects in brain plasticity when EEG devices are long term applied in developing children and adults (Tamburrini and Mattia, 2011). They also fear the unknown reversibility of these side-effects if the wearable is removed. However, no conclusive results have been appointed in this area.

Non-medical safety issues are more extensively mentioned and addressed in the literature. In this case, authors suggest that intense training and cognitive concentration could lead to potentially serious harms for EEG users, particularly in communication and control contexts, where cognitive planning and attention can lead to frustration (Glannon, 2014). Also, the need for regular and challenging training sessions may impose physical, emotional, and financial burdens on the users and their family (Fenton and Alpert, 2008). In addition, as users become increasingly dependent on technology, device failure and errors can similarly place users in dangerous situations (Hildt, 2011). This could endanger the life of its users in specific contexts. For example, an EEG wheelchair failing as its user is crossing a street or an EEG driven car causing an accident could lead to fatal consequences.

Another non-medical concern in this area relates to the vulnerability of commercial applications of BCI. Here, it has been demonstrated that deliberately-designed tiny perturbations templates for target attacks can manipulate EEG-based BCI speller to output anything the attacker wants with high success rate (Zhang et al., 2020). If these attacks may be targeted in other scenarios such as automatic driving, wheelchair control, or exoskeleton control, where the feedback plays an important role and the cost of one step mistake can have a big impact on the user, the security of EEG commercial applications should be reconsidered before deployment.

Since EEG is regarded as an inherently risky technology, which may lead to negative social outcomes, further studies should be conducted to clarify the acceptable expectations of benefit and risk. Nonetheless, several scholars suggest that this analysis may not yet be possible due to scientific uncertainty and lack of validated knowledge (Haselager et al., 2009).

### Agency

Agency is understood by ethicists as the ability of the individual to choose its own actions. The agency problem is frequently discussed by scholars from a two-fold perspective (*N* = 28). On a positive note, assistive technologies could lead to an increased agency via empowerment. On a negative note, the use of BCI could lead to an impairment of self-determination.

As an empowerment tool, several authors concur that EEG assistive technologies will allow patients greater independence (Mercer and Trothen, 2014; Zehr, 2015). This may enable patients to express their thoughts and behaviors, as well as to interact more independently with their environments, thus leading to higher standards of autonomy and human dignity. The increased agency conception acquires a greater significance in life-sustaining care contexts, where the informed consent plays an important role in end-of-life decision making. In this area, Glannon suggests that a weaker threshold version of understanding in decision-making about care would be justified for minimally conscious patients with a higher level of awareness and cognitive function who can clearly express their preferences about life-sustaining care through BCI-mediated binary responses (Glannon, 2016). This posture should be confronted with the general consensus in medical ethics about a high level of understanding for life-sustaining treatment with high probability of death (Drane, 1984; Appelbaum and Grisso, 1988; Jox, 2013; Peterson et al., 2013).

As an impairment tool, the actual threat to the social acceptance of mind-reading technologies lies in their potential capacity to “understand” consumer decision-making processes. This debate has been extensively addressed by public policy and academia, especially in the field of neuromarketing (Murphy et al., 2008). The scientific community is openly divided between researchers and practitioners who welcome this field (Garcia and Saad, 2008; Perrachione and Perrachione, 2008) and its detractors, including the general media (Blakeslee, 2004; Arussy, 2009). In most cases, the assumptions of these discussions have an economic substrate. As neuroscience and behavioral economics are proving to challenge “rational consumer” theories and their rational spending patterns, new approaches to the importance of marketing and advertising are emerging. In this sense, the use of neuroscience to understand the subconscious minds of consumers and, eventually, to alter their purchase decisions is an ethical concern widely disseminated in the literature (McDowell and Dick, 2013). Some authors believe that by measuring consumer's brain activity and developing effective communication techniques, corporations will be able to discover the “buy button” in consumers' brains. Thereby, they will be able to learn how to better trigger consumers' attention, which may ultimately lead to unprecedented levels of manipulation (Kelly, 1979; Wilson et al., 2008).

### Identity

The concept of identity is overarching in the literature as most scholars think that neurotechnologies could clearly disrupt the physical and mental integrity of the individual (*N* = 24).

In essence, the main concerns about identity are raised by invasive BCI. For instance, several studies have reported personality or behavioral changes leading to impulsivity, hypersexuality, mania, and gambling (Agid et al., 2006; Gisquet, 2008; Glannon, 2009). Alienation and estrangement have been also recounted in various treatments, where patients have stated that they “fe[lt] like a robot”, “an electric doll” or as if they were under “remote control” (Schüpbach et al., 2006; Goering et al., 2017). However, these changes do not occur in all cases and its origin still remains unclear in the scientific community (Johansson et al., 2014; Goering et al., 2017). As mentioned above, although these statements derive from neurosurgical interventions, the question remains open as to whether similar results could be envisaged when EEG is applied for motor recovery or permanent motor replacement. From what has been identified, there is an absence of preliminary research and pronouncements in this area.

### Enhancement

EEG permits direct communication between brains and computers. In the current state-of-the-art, EEG is clearly in an early stage of development for prediction, diagnosis, and restoration of functions, as previously mentioned in section Commercial Aspects. Notwithstanding, as technology advances, future qualitative leaps could allow superior functional enhancement (*N* = 21) (Hildt, 2011). This progression raises a number of questions about the nature of the human being.

As soon as people begin to incorporate their body schemes with enhancing neurotechnologies, which may allow them to radically expand their endurance and their sensory and mental capacities, authors like Hildt suggest that the notion of human being could be disrupted (Hildt, 2011). In particular, the extension of human limitations beyond the normal takes the debate on an ethical realm in which BCI users could become “cyborgs.” To this extent, Zehr believes that the development of sophisticated technologies that greatly enhance human intellect and physiology could transform the human condition (Zehr, 2015). As a result, *homo sapiens sapiens* could overcome its limitations and evolve into a *homo sapiens technologicus*, who takes advantage of the technology to improve its functioning (Zehr, 2015). This reassessment of the entire human predicament, as traditionally conceived, has been called “transhumanism” (Bostrom, 2005), and it is likely to change social norms, raise concerns about equitable access, and generate new forms of discrimination (Mercer and Trothen, 2014). Dystopian settings such as a class society, in which humans would coexist with enhanced humans, may come to fruition and create social stratification (Vlek et al., 2012). Likewise, nullifying equal access to resources as a consequence of unequal access to technology can aggravate social competence and unfairness among co-workers, thus generating new forms of discrimination (Kein et al., 2015). Of course, not all authors are convinced that these concerns are exclusive to BCIs or even possible. However, they arise with new vitality by virtue of advanced EEG-based BCI (McGee and Maguire, 2007).

### Privacy and Data Protection

The potential widespread use of EEG wearables raises a final set of issues that cluster around research ethics and the law (*N* = 15). As new ways of connecting to the brain emerge, new potential violations of user privacy might flare up. As enshrined in the General Data Protection Regulation (GDPR), which entered into force in 2018, brain data qualifies as sensitive data, thus triggering higher protection standards than those for personal data. This means that the grounds for processing sensitive data under the GDPR have become stricter and should comply with higher security standards. The legal basis of these restrictions is found in the greater impact that the misuse of such information could have on the life of the individual. As previously introduced in section Commercial Aspects, EEG devices could reveal a variety of information about the natural person, ranging from health and mental diseases and disorders, to psychological traits and mental states, creating potential problems such as discrimination based on neural information (Vlek et al., 2012). Indeed, some scholars suggest that because the EEG is capable of directly extracting sensitive information from the brain, a subject may be “unaware of the extent of information that is being obtained from his or her brain” (Vlek et al., 2012). Therefore, authors such as Farisco et al. note that in the biomedical sector, informed consent must respect (1) the disclosure of all needed information, (2) the capacity to understand it, and (3) the voluntariness to undergo the treatment (Farisco et al., 2015). In addition, in EEG-based commercial applications, companies and manufacturers must obtain a valid consent of the consumer by providing specific, informed and unambiguous information on the processing of his sensitive data. Some of the data that must be provided according to the GDPR are the identity of the controllers, the purpose of the processing, and the processing activities which may be carried out.

With regards to the processing activities to be carried out, a second privacy-related concern is the management of the extracted information. Here, scientists and the private sector have different interests. On the one side, the purpose of scientists is to uncover the objective truth and bring it among the public. On the other side, companies reluctantly give away their know-how as this is part of a highly competitive environment driven by profit maximization goals (Stanton et al., 2017). At this point, major ethical concerns arise when it comes to the sharing of this confidential data (Flores et al., 2014). The legislation on privacy and data protection guarantees that the information extracted will be kept confidential in a database, and its results should only be shared on scientific grounds and in an anonymous way to ensure the privacy of the research subject (Slowther and Kleinman, 2009). Failure to maintain the privacy of this sensitive data will be considered a violation of any ethical research practice and should result in the appropriate legal sanctions. Under the current European framework for privacy and data protection, violators can be fined up to 4% of their global turnover, or € 20 million.

Although there have not yet been any major scandals in the European Union regarding the processing and sharing of brainwave datasets, it has been reported fraudulent sharing of scientific research output in the United States. Consumer groups have claimed that Emory University and Baylor School of Medicine violated the Belmont Report's principle of beneficence -which entails an obligation to protect individual subjects against risk of harm and the societal benefits that might be gained from the research- by partnering with neuromarketing companies (Fisher et al., 2010; Pop and Iorga, 2012; Ulman et al., 2015; Stanton et al., 2017). This situation has led to the modification of national legislations worldwide. For instance, France, which faced protests against neuroscience research, banned the use of brain-imaging methods for commercial purposes in 2011. The government argued that the processing of consumer brain signals might constitute an invasion of privacy and they should be solely processed for medical or scientific purposes (Ulman et al., 2015).

Taking all these facts into consideration, privacy and data protection are deemed as extremely important aspects to be considered for a peaceful transition into a BCI society.

## Discussions and Conclusions

The present scoping review provides a holistic view of EEG-based market applications, as well as identifies the most relevant ethical questions arising from the existing literature. Yet before discussing these issues, several limitations of this study should be considered. Although the study was conducted using different databases, most of the articles found were solely based on biomedical applications of BCI. By the same means, the ethical debate revolving around neural technologies was thus primarily focused on a biomedical approach. We consider that the limited number of articles that evaluated EEG from other non-biomedical research domains hampers the purpose of the present review. In order to draw more precise conclusions about the subject-matter of this study, more research should be conducted from a non-biomedical scope. This may emphasize different ethical connotations and present this technology through alternative methodological lenses. More broadly, we also identified that most of the ethical considerations were asserted in a general BCI context, specially by taking into consideration invasive BCI. Despite the fact that invasive and non-invasive methods might foreseeably share a large number of identical ethical concerns, more scientific effort should be made on specific non-invasive risks in order to legitimize the discourse surrounding EEG applications. Another limitation of this review is that most of the literature addresses the ethical problems from an advanced technology-based perspective instead of focusing on the present state-of-the-art. Since one the main duties of ethicists is to anticipate the new scenarios and living conditions implicit in the relentless progress of technology, we are prone to think that this approach may enhance the existing technological capabilities and provide a distorted view of reality. For this reason, we consider that the ethical findings obtained in this review might be treated with a small dose of scientific skepticism. Furthermore, the present review addresses only those issues that were recurrently cited across the coded articles with brief reference of other seldom detected topics. Commercial applications and ethical problems that were rarely cited, though underrepresented in this review, may be just as significant as the categories described above. Despite these limitations, there are several features of the literature sample that can be highlighted here.

### Commercial Aspects

There is no doubt that the overlap of science and markets is inevitable, and EEG has the potential to revolutionize these spaces. As stated, the applications of EEG are wide and can be employed in different sectors and industries. Hitherto, EEG solutions have been mostly explored in the medical sector for prediction and diagnosis of various health conditions, as well as for treatment, rehabilitation and assistance (*N* = 74). They have been used as a rehabilitation tool for motor recovery after spinal cord injury, as spellers for individuals who have no other way to communicate; and as a means to control the environment of people who are locked-in or paralyzed. Other industries in which EEG has been successfully implemented are neuroergonomics, smart tracking devices, video games, neuromarketing, education, and authentication systems, among others. However, before EEG becomes widely accepted as a useful and reliable tool in the commercial sphere, several shortcomings need to be corrected.

First, EEG may be technologically questioned, so it should be treated with a degree of caution given the actual limitations of the current state-of-the-art. Secondly, for EEG technologies to be mass marketed, some breakthroughs must be found. Thirdly, from a cost-benefit standpoint, EEG may not be the best option to invest at the moment due to its minimal commercial viability. Fourth, for EEG to become satisfactory end-products, they should also focus on a user-friendly design.

### Limitations of EEG

Although a considerable amount of experimental evidence supports the notion that EEG techniques can provide relevant insights into the dynamic processes of the brain, authors also point out that their numerous benefits could be questioned (Lopes Da Silva, 2013). Currently, neuroscience research is limited not only by revealing what is occurring in the brain, but also by explaining why it occurs, thus making reliability a difficult aspect to improve. It should be noted that EEG is still a relatively new technology and further advances should be made before the dynamics of the cognitive processes are completely unraveled.

As far as consumer EEG devices are concerned, they may be additionally distrusted due to several restraints. For instance, Leitão & Campos argue that the number of electrodes on consumer wearables is limited compared to the clinical-grade devices. Also, the electrodes are usually focused on a specific area of the brain and their resolution is lower compared with those of high-clinical density (Leitão and Campos, 2017). Swati et al. discuss that any eye movement, muscular activity or electronic devices in the vicinity of such commercial devices introduce artifacts to the signal that can disrupt the measurement of actual brain waves (Vaid et al., 2015). They also claim that numerous possible features with minimal computation and over-specification are still a key problem when considering recognition performance of the signal (Vaid et al., 2015). Spapé et al. recognize that the validity of classification algorithms for commercial EEG applications cannot be confidently assessed due to brand opacity and trade-secrets (Spapé et al., 2015). Finally, Poldrack suggests that the inferential methods used to study cognitive processes, which may be predictable to some extent, could be unreliable with respect to reverse inference (Poldrack, 2006). This, alongside with the low signal-to-noise ratio (SNR) of consumer consumer-grade devices, questions their validity as reliable neuroscience solutions.

Since EEG should then be regarded with some degree of doubt and the future of this technology is still uncertain, attention and scientific rigor shall be applied when formulating proposals about its future potentials. As several authors have already pointed out, a greater debate should be generated on the real effects of mind-reading technologies in order to prevent the naïve misconceptions that the information media can instill in the general public (Kenning, 2008; Weisberg et al., 2008; Spapé et al., 2015). These false assumptions about the objectivity and trustworthiness of consumer neuroscience solutions visibly magnify their real capacities, which are still far from their forecasts. Indeed, actual consumer-grade devices may well-satisfy consumers, but from a scientific point of view their reliability and effects still remain unclear.

### Technological Breakthroughs

Even if the natural limitations of EEG could be remedied, there still remains the need for technological disruptions to ensure the reliability of the device. As part of this approach, a growing body of evidence shows that EEG is clearly observing an asymptotic trend in the accuracy for cognitive state or intent estimation that converges to a significant error rate of 5–20% depending on the targeted cognitive variable (Makeig et al., 2012). It is possible that this trend may not be significantly curved by the sole action of incremental improvements alone. Disruptive innovations should take place in order to allow for the scaling up of both the amount of integrated information and the amount of offline and online computing (Kurzweil, 2001). For example, such innovations may arrive in the form of better electrophysiological sensor technologies, possibly via extremely high channel-count and signal-to-noise-ratio non-invasive systems. The combination of these systems with sufficient computational resources could conceivably allow the modeling of brain activity at a range of spatial-temporal scales, as well as considerably reduce measuring errors, as postulated by Vaid et al. (2015). Additionally, to reach this level of information density, safer and more efficient procedures should be developed to enable closer-to-brain source measurements (Liao et al., 2012; Hoodgar et al., 2013). Thus, for EEG wearables to become as useful as computer mice and touch screens, technological breakthroughs are required that exceed the marginal improvements of current information processing techniques. Recent entrepreneurial initiatives are attempting to achieve these goals. In the field of communication and control, Facebook's purpose is to accelerate mobile device communication using non-invasive techniques to reach brain typewriting speeds of 100 words per minute (Strickland, 2017). However, at the present time there is still no pronouncement on the success of this type of initiatives.

### Cost-Benefit Trade-Offs

Aside from the limitations and technological breakthroughs that have been highlighted, another commercial issue that needs to be examined is the economic viability of EEG solutions to become satisfactory consumer-grade devices.

As previously mentioned, the present scoping review has determined that medical applications of EEG represent its most prominent uses. Indeed, EEG has been used as an assistive technology in the biomedical realm by default. The fact that EEG is mostly applied in the medical field and its solutions are primarily targeted at particular users, such as people with motor disabilities and LIS patients, has a high impact on its business prospects. This user population barrier has already been recognized by several authors, who believe that the high specificity of EEG users limits the market niche to which EEG solutions are directed (Nijboer, 2015). Having thus a reduced number of users, the market entry barriers for EEG investment are relatively high, and the expected reimbursement for commercial commitment tends to be low, as suggested by Nijboer (2015). This is the fundamental reason why BCI development is at the moment primarily subsidized by the European Commission (€ 11 million in FP6 and € 34 million in FP7), as its dimensions are not likely to attract the attention of the industry (Nijboer, 2015).

Despite this fact, as EEG-based pBCI applications begin to be deployed for purposes other than traditional aBCI applications, i.e., they start to be used as smart tracking solutions in diverse sectors such as those identified in the literature, their attractiveness could be increased. Consequently, novel EEG applications may expand their initial tailored group to wider user populations, thus increasing investment in EEG.

### User-Friendly Design and Experience

Finally, for EEG to become satisfactory end-products, they should also focus on design for usability. As some authors have stated, most of the current EEG prototypes are evaluated on the basis of speed and accuracy, rather than on usability (Moghimi et al., 2012), and they have argued that EEG engineers should integrate ergonomic factors and human-computer interaction principles into the design of their products (Bos et al., 2010; Pasqualotto et al., 2012). Here, one of the most critical concerns for EEG wearables is the inconvenience to wear them in large-scale samples for extended periods of time (Xu and Zhong, 2018). If these problems could be solved in the near future, for example, by building more adaptive and portable wearables, EEG would be more widely used. In the same vein, the aesthetics of the device may be as important to users in everyday life, if not more so, than the technology itself. These can be key factors for the success of EEG products and services.

Furthermore, consumer experience is also an issue that has been extensively addressed. At an individual level, the use of EEG in sectors such as self-regulation frequently becomes either boring or frustrating over time (Nafus and Sherman, 2014). When, however, it is elevated to a collaborative level, some authors postulate that better results are achieved (Lupton, 2013). Most likely, the future of EEG-based pBCI lies in collaborative endeavors. Since human beings are inherently social creatures, advanced EEG technologies could foster their interactions. For example, consumers could be empowered and motivated by bringing them into large-scale interactive projects or programs in which users may communicate within the legal constraints. In addition, the user experience may be greatly improved by detecting users' affective states to adapt individual and collaborative features. In this sense, by mobilizing a new generation of EEG headsets focused on user-friendly experience, this technology could be brought on board pervasively (Swan, 2016).

### Ethical Aspects

Overall, the results of this review show that EEG sparks concern over many ethical problems and questions that should be addressed, particularly in the literature of biomedical ethics. It seems that further attention on the social impact of neural technologies in the following fields should be paid.

### Safety and Risk-Benefit Analysis

The most frequently cited problem in the literature concerns safety in medical and non-medical settings (*N* = 33).

In the first case, it has been noted that there is lack of literature handling this concrete ethical problem for non-invasive applications such as EEG. Although there is a predominance of discussion on the potential negative side effects in brain plasticity of EEG wearables, the results are inconclusive and the hazards have not yet been validated. Given that EEG technology is currently being assessed without direct acknowledgment of the above concern, ethicists deliberating on this topic may need to wait until more robust conclusions about the real side effects of long-term uses of EEG devices are presented. Nevertheless, as these devices are easily accessible over the internet and can be expected to be worn ubiquitously in the future, medical hazards remain important issues for which some consideration must be given.

In the second case, non-medical risks, such as frustration, have been broadly mentioned in the literature as problems that justify improvements in EEG wearables. These advances could come in the form of psychologically adaptive EEG. Such devices may interact with user mental states, leading to a reduction in training frustration and in users' physical and emotional burdens. Several authors have proposed overt adaptive EEGs that would be automatically deactivated when extremely low attention levels are detected or reactivated when the user's attention has returned (Fairclough, 2009). It has also been suggested the development covert adaptive devices which would autonomously modify their classification algorithm to adapt to changes in the users' mental states (Fairclough, 2009). This would allow more sensible and dynamic wearables as well as an increase of user experience. Device failure and algorithmic vulnerability are also critical issues highlighted by several authors. In these cases, we expect improvements to arrive as soon as technological breakthroughs appear, which allow EEG processing capabilities to be more robust and precise as well as more cybersecure.

Since safety is thus a critical issue, more extensive high-level discussions should be held on the relative risks and benefits of EEG devices. These discussions should highlight the importance of elaborating risk-benefit assessments by comparing EEG solutions with alternative assistive technologies, as stated by some authors (Tamburrini, 2014).

### Agency

There are a remarkable number of articles in the literature dealing with the ethical implications of agency through the use of neural technologies (*N* = 28). Here, the main highlighted issues were related to the increased/decreased agency, as mentioned above.

Regarding the positive effects of EEG, authors suggest that the possibility of increased agency via EEG assistive applications is one the most important benefits of this technology. Here, the empowerment of functionally diverse individuals has been extensively addressed, and there is agreement about the decrease of social stigma that neural technologies may lead to. As neural technologies start being included in patients and consumers daily-life activities, they may enhance their lost agency capacity as well as return them the necessary confidence to autonomously interact with their environment. This can be of great relevance in contexts where patients' autonomous decision-making may enable them to achieve higher standards of living and dignity, especially in end-of-life situations. Regarding the negative effects, it has been identified that the incorporation of EEG technologies into the body-schemes of consumers could lead to several downsides, such as new forms of manipulation which may limit user's agency.

Bearing in mind the trade-offs of EEG, the key issue that remains to be identified is patient's preferences. It is essential to balance the interference and support of EEG systems in the user's daily activities. Thus, it could be possible that users may be willing to sacrifice some level of exposure in favor of having a greater capacity to do what they want to do or not. Understanding what would count as a reasonable balance for the user must be part of the design process, and it might seem that a system that offers options to the user would be preferable (Gilbert, 2015; Hoppe et al., 2015). This may have a profound impact on the incorporation of EEG assistive tools into the “body schemas” (Heersmink, 2013) and “structures of decision-making and acting” of patients (Clausen, 2008), as well as on the deployment of EEG in the consumer markets. In any case, the practical implications of the agency debate are of the utmost importance, as they could challenge the social acceptance of this technology.

### Identity

In contrast to the agency issue, the identity problem has been less extensively validated through the literature. Notwithstanding, there are still a notable number of articles in the literature dealing with the ethical implications of identity (*N* = 24). These concerns are eminently raised in invasive contexts of use. Here, more empirical research should be conducted in the area of non-invasive devices in order to draw well-founded conclusions. Beyond this preliminary debate, it should be further examined whether these types of changes should be understood as threats to identity *per se* or as simple alterations that may be beneficial or detrimental to the individual (Schermer, 2009; Baylis, 2013). To this extent, some scholars have already pointed out that the interpretation of the concept of identity as something fixed or mutable over time constitutes the premise of this debate (Goering et al., 2017). Therefore, a broader debate about the concept of identity should be conducted, as well as it should be evaluated *ex ante* whether or not these changes in personal identity may be a real problem that could have an impact on technological development and access to EEG.

### Enhancement

Enhancement through neural technologies poses several concerns as identified in the literature (*N* = 21). We consider that these propositions should be treated from a broad perspective and, in any case, as long-term assumptions. Indeed, research shows that the technology stage is still premature to perceive the ethical implications of augmentation as present real-life problems. What should be noted is that public engagement, ethical deliberation, and legal frameworks shall be developed in order to accomplish a peaceful transition toward ubiquitous EEG. Since the ultimate goal of scientific research is social welfare, the deployment of neural technologies should obey the ethical and legal standards agreed upon by society. Therefore, involving the public in the debate and discussion on new emerging technologies is an essential requirement for this transition. Particularly, actions should be undertaken to inform, educate, and shape public policy regarding the use of neural technologies. As Heidegger indicates, the key to transitioning to a future of greater human-technology integration in an empowering manner is to consider the public opinion and tolerance, as well as to maintain “the right relationship with technology” (Heidegger, 1997). This relationship is an interaction in which technology enables but does not enslave.

### Privacy and Data Protection

Lastly, the findings of this review indicate that with the deployment of EEG wearables as consumer-grade devices, large amounts of sensitive data about the data subject will be collected and processed (*N* = 15). Consequently, private and public entities shall ensure transparency in relation to the processing of these pieces of data, as well as the appropriate security and confidentiality of the personal data relating to the data subjects. In addition, institutions shall stay updated to avoid any possible data breaches, and develop strong cyber hygiene practices and secure products. For example, communications between wearable sensors and processing or storage units shall be based on encrypted protocols to ensure appropriate security levels. Likewise, firewalls and domain name server-based security solutions should be kept updated to prevent unauthorized access and protect devices when they are exposed in everyday contexts.

On the other hand, any entity that keeps sensitive information will have to engage with industry and government standard's bodies to establish and steward technology norms as features. This would entail safe and secure processes, including a consent procedure that clearly specifies who will use the data, for what purposes and for how long. Consumers and patients should be assured that information and results concerning them will be kept confidential in a database, and that results shall be shared only on scientific grounds and anonymously to maintain their privacy rights.

## Data Availability Statement

The original contributions presented in the study are included in the article/Supplementary Material, further inquiries can be directed to the corresponding author/s.

## Author's Note

Certain legal implications of SECTION IV, *D. Privacy and data protection*, were not part of the scoping review and are provided for explanatory purposes only.

## Author Contributions

CF made the literature review and wrote the paper. GL edited the paper. DZ is the supervisor, who conceived this study and edited the paper. All authors contributed to the article and approved the submitted version.

## Conflict of Interest

The authors declare that the research was conducted in the absence of any commercial or financial relationships that could be construed as a potential conflict of interest.
